# Unravelling the intertwined atomic and bulk nature of localised excitons by attosecond spectroscopy

**DOI:** 10.1038/s41467-021-21345-7

**Published:** 2021-02-15

**Authors:** Matteo Lucchini, Shunsuke A. Sato, Giacinto D. Lucarelli, Bruno Moio, Giacomo Inzani, Rocío Borrego-Varillas, Fabio Frassetto, Luca Poletto, Hannes Hübener, Umberto De Giovannini, Angel Rubio, Mauro Nisoli

**Affiliations:** 1 Department of Physics, Politecnico di Milano, 20133 Milano, Italy; 2 Institute for Photonics and Nanotechnologies, IFN-CNR, 20133 Milano, Italy; 3 Center for Computational Sciences, University of Tsukuba, Tsukuba, 305-8577 Japan; 4 Max Planck Institute for the Structure and Dynamics of Matter, 22761 Hamburg, Germany; 5 Institute for Photonics and Nanotechnologies, IFN-CNR, 35131 Padova, Italy; 6 Nano-Bio Spectroscopy Group, Universidad del País Vasco, 20018 San Sebastian, Spain

**Keywords:** Ultrafast photonics, Electronic properties and materials

## Abstract

The electro-optical properties of most semiconductors and insulators of technological interest are dominated by the presence of electron-hole quasi-particles, called excitons. The manipulation of excitons in dielectrics has recently received great attention, with possible applications in different fields including optoelectronics and photonics. Here, we apply attosecond transient reflection spectroscopy in a sequential two-foci geometry and observe sub-femtosecond dynamics of a core-level exciton in bulk MgF_2_ single crystals. Furthermore, we access absolute phase delays, which allow for an unambiguous comparison with theoretical calculations. Our results show that excitons surprisingly exhibit a dual atomic- and solid-like character, which manifests itself on different time scales. While the former is responsible for a femtosecond optical Stark effect, the latter dominates the attosecond excitonic response. Further theoretical investigation reveals a link with the exciton sub-femtosecond nanometric motion and allows us to envision a new route to control exciton dynamics in the close-to-petahertz regime.

## Abstract (web summary)

The capability to follow electron motion in solids is necessary to explore the ultimate speed limits of optical charge manipulation and signal processing in optoelectronic devices. Here, the authors reveal the sub-femtosecond dynamics of core excitons in MgF_2_ and find the dual atomic-solid nature of the exciton quasi-particle to deeply affect its ultrafast dynamics.

## Introduction

The quest for new devices capable of surpassing the current technological limits^1^ has pushed the scientific community to explore solutions beyond classical electronics as done in excitonics, spintronics and valleytronics^2^. Therefore, studying the dynamics of excitons in solids^3–5^ becomes a priority task not only to widen our knowledge of fundamental solid-state dynamical phenomena, but also to explore the ultimate limits of these novel technologies^6–11^. While the development of attosecond spectroscopy^12^ has proven the possibility to study sub-femtosecond (fs) electron dynamics in solids^13^, shedding light onto strong-field phenomena and light-carrier manipulation^14–16^, a clear observation of attosecond exciton dynamics was missing. Besides more conventional femtosecond techniques^17^, attosecond transient absorption and reflectivity spectroscopy have been employed to study the ultrafast decay processes (few-fs) of core-excitons^18,19^, but failed in recording the sub-cycle dynamics unfolding during light–matter interaction. Here we used attosecond transient reflection spectroscopy (ATRS)^20^ to study attosecond dynamics of a core-level exciton in bulk MgF_2_ single crystals, a widely used material in optics, characterised by a clear core^21^ and valence^22^ exciton signal which make it an optimal target to investigate exciton dynamics with attosecond techniques. Thanks to the employment of simultaneous and independent calibration experiments, we achieved direct comparison with theoretical simulations, which in turn allowed us to make a clear link between the observed transient features of the system optical response and the nanometric and attosecond motion of the excitons. Holding in general for Wannier-Mott excitons, including valence excitons, our results provide a description of the ultrafast exciton–crystal interaction on sub-fs time scales and move an important step towards a more complete understanding of attosecond light-excitonic dynamics.

## Results

**Fig. 1:**
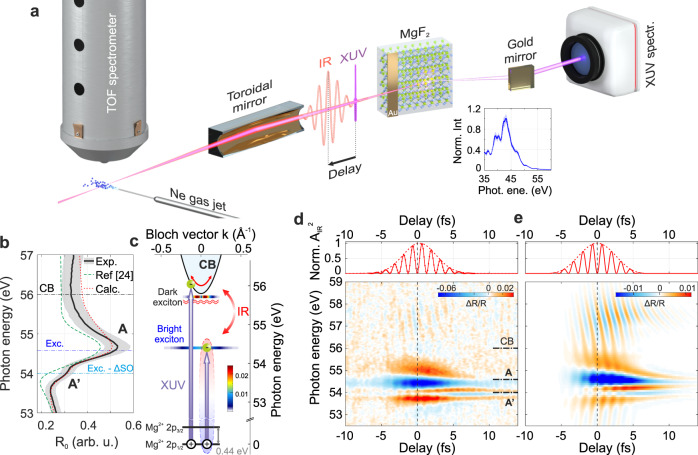
MgF_2_ core-exciton optical response. **a** Scheme of double-foci the experimental setup composed by the Ne gas target to perform an attosecond streaking experiment, the MgF_2_ crystal and the XUV spectrometer. The inset shows the attosecond pulse spectrum as reflected by the MgF_2_ sample. **b** Experimental (black solid) and calculated (red dotted) MgF_2_ reflectivity for an incidence angle of 73.5°. The grey shaded area represents twice the standard deviation over repeated experimental measurements, while the green dashed curve is the reflectivity extracted from Hanson et al.^24^. The horizontal dash-dotted lines mark the vertical transitions from the Mg^2+^ 2*p* core level to the conduction band (CB) and the bright exciton^21^, accounting for the spin–orbit splitting of the Mg^2+^ 2*p* state. **c** MgF_2_ band structure around its CB. While XUV photons can promote electrons from the Mg-2*p* state directly into the CB, or to an electron–hole pair state (exciton), the IR field perturbs the crystal, causing phenomena like intra-band motion or dressing of dark excitonic states. The false colours represent the density of states for the excitons. **d** Experimental and **e** calculated transient reflection spectrograms (main panels) as a function of the delay between the XUV and IR pulses, together with the square of the IR vector potential, *A*_IR_^2^, as extracted from the simultaneous streaking measurement (upper panels). The delay zero (vertical dashed line) has been chosen in order to coincide with a maximum of the IR electric field squared. The experimental IR peak fluence is 0.08 J/cm^2^.

Figure 1a shows a schematic picture of the experimental setup characterised by a sequential two-foci geometry, used to perform simultaneous attosecond photoelectron and ATRS measurements^23^ (see Methods). The static reflectivity for a MgF_2_ (001) crystal, *R*_*0*_, close to the Mg L_2,3_ edge is probed by an extreme-ultraviolet (XUV) attosecond (as) pulse (Fig. 1b). In this energy region *R*_*0*_ is characterised by a peak (labelled with A), which has been attributed to the formation of excitons after excitation of a Mg^2+^ 2*p* core electron^24^ (Fig. 1c). The weaker satellite peak at about 54 eV (A′ in Fig. 1b) originates instead from the spin–orbit splitting of the Mg^2+^ 2*p* core state^25^. In our experiment we use a 5 -fs infrared (IR) pulse (centre wavelength 750 nm, peak intensity 10^12^–10^13^ W/cm^2^) to drive the crystal out of equilibrium (Fig. 1c). The induced ultrafast exciton dynamics are probed with 250-as pulses by monitoring the sample differential reflectivity Δ*R*/*R* in the XUV range, defined as the difference between pumped and unpumped reflectivity, divided by the latter: [*R*_IR_ − *R*_0_] /*R*_0_. The results as a function of photon energy *E* and pump-probe delay *t*, are shown in Fig. 1d. At small values of *t*, we observe rich transient features which unfold either on few-fs or attosecond time scales (hereafter referred to as slow and a fast component, respectively). While the former is mainly located around the excitonic features A and A′, the latter oscillates at twice the IR frequency and extends over the full energy range under consideration, becoming more evident in the conduction band (CB) region. The weak differential signal already present at negative delays around 54.4 eV originates from a non-vanishing IR electric field. It goes below the experimental noise level for delays smaller than −15 fs and does not influence our analysis (see Supplementary Note 6). The upper panel in Fig. 1d shows the square of the IR vector potential, *A*_IR_^2^, as retrieved from the simultaneous streaking trace (see Supplementary Note 7). Knowing the associated pump field time evolution, we calibrated the pump-probe delay axis in order to have the zero delay coinciding with the maximum of the IR electric field squared (equivalently, a zero of *A*_IR_^2^). This allowed us to set an absolute reference for our measurements and study the precise timing of the system dynamics.

To reach a complete understanding, we calculated the quantum dynamics with a Wannier-Mott (WM) exciton model^26,27^ (see Methods) and chose the same reference for the delay zero, such that we could directly compare experimental and theoretical results. The calculated Δ*R*/*R* reported in Fig. 1e accurately reproduces the experimental data of Fig. 1d, suggesting dynamics which go beyond the optical Stark effect^28^. While the calculated and experimental Δ*R*/*R* fall in the same order of magnitude, there are appreciable differences in their absolute values which may originate mainly from an underestimation of the pump intensity. In addition, we observe that the calculations overestimate the oscillatory part in the CB with respect to the slow component. This may originate from a combination of unavoidable experimental imperfections (any instability will reduce the contrast of the fast oscillation) and the approximations made in our theoretical model. The observed discrepancies have anyway no effect over the Results and Discussion reported in this paper.

## Discussion

**Fig. 2:**
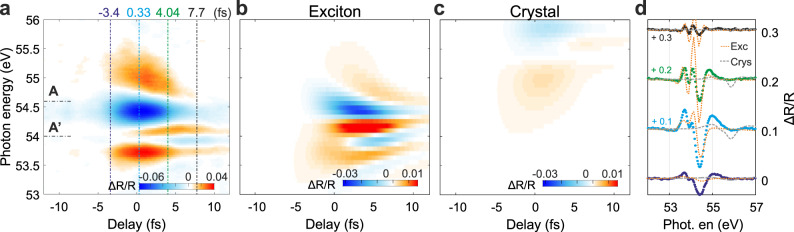
Real-time observation of femtosecond core-exciton dynamics. **a** Slow component of the experimental Δ*R*/*R* reported in Fig. 1d. **b**, **c** Calculated slow component of Δ*R*/*R* considering solely the two excitonic states or only the crystal CB, respectively. **d** Measured (dots) and calculated (orange dotted and grey dashed curves) Δ*R*/*R* profiles for four representative delays marked by the dash-dotted vertical lines in (**a**).

The slow component of the experimental Δ*R*/*R* (Fig. 2a) is characterised by a series of positive (red) and negative (blue) features, which develop around delay zero and fully disappear within 10–15 fs. These features originate mainly from IR-induced optical Stark effect of the excitonic transitions, and the presence of dark excitonic states which become optically active around zero pump-probe delay^19^. The exciton optical Stark effect is the analogous of the well-known optical Stark effect in atoms where absorption or emission of non-resonant photons can lead to a shift of the atomic energy levels^29^. In analogy with a photon-dressed two-level system, the optical Stark effect originates from the coupling between the bright and dark excitonic states. Since the photon energy (~1.6 eV) is bigger than the spacing between the two states (~1.3 eV), the associated transition is red-shifted, which translates in a blue shift of the Mg 2*p*—bright exciton transition.

Figure 2b, c shows the calculated slow component of Δ*R*/*R* considering only the excitonic states reported in Fig. 1c (“pure excitonic” contribution) or only the crystal CB (“pure crystal” contribution) (see Methods). We find that “pure exciton” calculations qualitatively reproduce the main experimental features while the crystal response does not (Fig. 2d).

Since in our pure-exciton model the exciton is described with atomic-like states, solid-like interactions with the IR field (e.g. intra-band motion) are not possible. Therefore, the good agreement between the calculations of Fig. 2b and the experimental data of Fig. 2a supports the attribution of the origin of the slow component of Δ*R*/*R* to the optical Stark effect and shows that the few-fs dynamics of the optical response can be understood, on first approximation, by considering only the atomic-like character of the exciton quasi-particle.

From the delay-dependent optical Stark shift, it is possible to study the exciton decay process^19^. We found the bright exciton to decay rather quickly with an effective time constant of 2.35 ± 0.3 fs (see Methods), comparable to what observed for other insulators like SiO_2_^18^ and MgO^19^. A deeper analysis, which could reveal the interplay between Auger decay and phonon coupling, needs either measurements with a shorter IR pulse, or more complex reconstruction procedures, both of which go beyond the scope of this work.

**Fig. 3:**
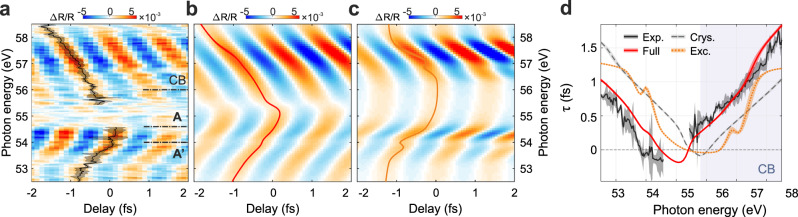
Core-exciton dynamics on an attosecond time scale. **a** Fast component of the experimental Δ*R*/*R*. The phase delay, τ, between the oscillations and *E*_IR_^2^ is displayed in black. The solid curve represents the mean over four independent measurements and the shaded area twice its standard deviation. **b**, **c** Same as (**a**) but for the full or the pure exciton calculation results, respectively. **d** Comparison between the experimental (black solid) and calculated τ, considering the full system response (red solid), or addressing separately the crystal (grey dashed) and excitonic (orange dotted) contributions. .

While the fact that some optical properties can be described by the exciton atomic character is typical for a quasi-particle characterised by a binding energy of 1.4 eV^21^, the analysis of the sub-fs dynamics reveals an unexpected result. The fast component of the transient reflectivity spectrogram is reported in Fig. 3a, showing clear oscillations at twice the IR frequency, which are fully reproduced by our simulations (Fig. 3b). Both the amplitude and the tilt of the oscillations cannot be reproduced considering solely the exciton contribution (Fig. 3c). These oscillations have a V-shaped dispersion which resembles what is found for the dynamical Franz-Keldysh effect (DFKE) high into the CB of diamond^30,31^, suggesting a clear link with intra-band motion of virtual charges. We note that the interaction regime significantly differs from DFKE in valence excitons exposed to THz pulses^32,33^ where the pump field evolves on a much slower time scale. Furthermore, even if the lower 2*p* state is non-dispersive, the energy of the electron–hole particle follows the upper CB state and exhibits a parabolic profile. This explains why a V-shaped structure is observed even when the initial state is not a dispersive valence state, but an atomic-like core level.

Thanks to our independent pump-probe delay calibration, we can make a further step towards a complete comprehension of such a rich exciton dynamics and study the phase delay τ between the oscillations in the transient signal and the square of the IR electric field *E*_IR_^2^ (see Methods). Figure 3d presents the experimental τ obtained as a weighted average over four independent measurements (black solid curve) compared with the calculated phase delay in case of full model (red solid curve), pure exciton (orange dotted curve) or pure-crystal response (dashed grey curve). In all cases, the shaded area represents twice the standard deviation originating from the measurement error or from the uncertainty of the phase extraction method. The full model accurately reproduces the experiment both on a qualitative and quantitative level. However, in contrast to what we observed for the slow component of Δ*R*/*R*, now the pure excitonic contribution alone fails in capturing the measured τ, even qualitatively. This strongly indicates that the fast component of the differential reflectivity is dominated by the solid nature of the exciton, despite its localised character and in stark contrast to its slower atomic-like response. Indeed, the oscillations found in the pure exciton model (Fig. 3c) originate from an atomic effect: the instantaneous optical Stark shift. On the contrary, the oscillations observed in the pure-crystal model arise from the intra-band motion of virtual carriers (DFKE). In principle, real carrier motion at the CB bottom cannot be neglected^31^. Nevertheless, as observed in GaAs crystals^21^, while the total carrier injection rate into the CB is considerably affected by the interplay of inter- and intra-band transitions, the phase of the ultrafast optical response is mainly dictated by the motion of the virtual carriers. Therefore, we conclude that the latter plays a major role in sculpting the energy dispersion of the oscillation phase reported in Fig. 3a. The fact that the full system response (red curve in Fig. 3d) resembles the bare crystal case (dashed grey curve in Fig. 3d) but shifted in energy, suggests the following physical interpretation. The few-cycle IR pulse dresses the crystal CB inducing virtual charges intra-band motion (DFKE). In turn, this alters the exciton dynamical properties causing the quasi-particle to oscillate in the IR field with a phase relation similar to that of the bare crystal.

**Fig. 4:**
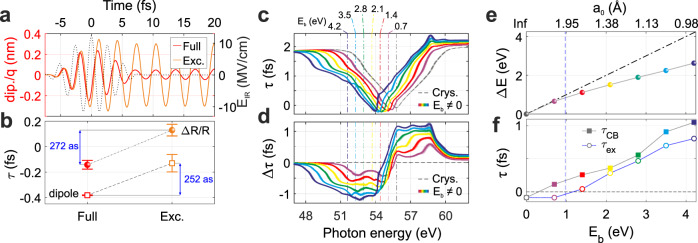
Exciton real space dynamics and role of localisation. **a** Exciton dipole oscillations in real time computed with the full (red) or pure exciton (orange) model. The black-dotted curve represents the IR electric field. **b** Comparison of the phase delay extracted from Δ*R*/*R* (circles) or from the dipole moment (squares) for the full calculations (red) or considering only the excitonic response (orange). **c** Behaviour of the calculated phase delay τ as a function of the exciton binding energy *E*_b_. The vertical dash-dotted lines mark the exciton position. **d** Difference between the phase delay of the full system and the pure-crystal response, Δτ, for the data reported in (**c**). **e** Energy shift Δ*E* which minimises Δτ as a function of *E*_b_ (Bohr radius *a*_0_). The vertical blue dashed line marks the minimum inter-nuclear distance in the MgF_2_ crystal unit cell equal to 1.98 Å. **f** Value of the phase delay τ evaluated at the exciton vertical transition (τ_ex_, open circles), or at the bottom of the CB (τ_CB_, full squares) for the different values of *E*_b_ (*a*_0_) considered.

If the proposed picture is correct, we expect the observed transient features in the optical response to correspond to an actual movement of the exciton on attosecond (as) and nanometric scales. To tackle this, we calculated the excitonic dipole in real time and found that it oscillates almost at the same frequency of the IR field during interaction. The results are shown in Fig. 4a for the case of the full system (red curve) or considering only the quasi-particle (orange curve). By evaluating the phase delay for the dipole with respect to the IR field (black dotted curve), we found the oscillations of the pure exciton dipole to be delayed by 252 ± 69 as with respect to the full system response (square marks in Fig. 4b). Remarkably, a similar shift of 272 ± 57 as is observed between the oscillations of Δ*R*/*R* evaluated at the energy of the excitonic transition and calculated with the full model or considering only the excitonic contribution (full circles in Fig. 4b). Therefore, our findings suggest a strong correlation between the sub-nm exciton motion in real space and the sub-fs transient features observed in the differential reflectivity. This not only proves that localised excitons can show properties beyond atomic model, but also opens the possibility to investigate separately the optical Stark effect (atomic-like) and the DFKE (solid-like), which were previously found to compete in time-averaged measurements^32^, widening our comprehension of the ultrafast solid-state physics^34^.

So far we have proven that exciton–crystal interaction can transfer bulk properties to atomic-like core excitons on attosecond time scale. All the more reason, the same is expected to happen for Wannier-Mott valence excitons which, being more delocalised, have a stronger solid-like behaviour (see Supplementary Note 19). The question remains whether the attosecond response will qualitatively change, deviating by the solid-like response, if more localised excitons are taken into considerations. In order to further investigate the role of real space dynamics and exciton localisation we computed the reflectivity phase delay τ for different exciton binding energies *E*_b_ or, equivalently, Bohr radii *a*_0_, which provides a reference for the actual size of excitonic devices^10,35^. With increasing *E*_b_ and thus the degree of localisation, τ preserves its V shape whose centre appears to move towards lower photon energies (Fig. 4c), resulting in an overall bigger phase delay difference, Δτ, between the bare crystal and full system responses (Fig. 4d). The energy shift Δ*E* which minimises Δτ is not exactly equal to *E*_b_ (Fig. 4e), indicating that τ does not simply experience a rigid shift following the exciton transition. On the one hand this further confirms that the central role of exciton–crystal interaction, on the other hand it opens a way to sculpt and control the excitonic response in the petahertz regime. While the optical response of delocalised electron–hole pairs will react almost in phase with the pump electric field (as underlined by the phase delay τ evaluated at the exciton vertical transition, τ_ex_ in Fig. 4f), more localised excitons will have a slower response, almost out of phase. Due to the exciton–crystal interaction, the CB response will also be significantly affected (see the phase delay τ evaluated at the CB bottom, τ_CB_ in Fig. 4f). Since the exciton binding energy can be continuously tuned around its natural value by an external field, modifying the dielectric screening or inducing a strain^36–38^, one can control the phase delay between the quasi-particle and an optical field on an attosecond level. In particular, for values of *E*_b_ ≈ 1 eV where *a*_0_ becomes comparable with the minimum inter-nuclear distance in the MgF_2_ unit cell, it is possible to control the attosecond timing between the exciton and the CB signal, realising a condition when the first is advanced with respect to the IR field while the second is delayed.

As the attosecond response of valence excitons behaves qualitatively in the same fashion (see Supplementary Note 19), our findings point to a possible route for the realisation of a different class of devices where a control over the degree of Coulomb screening^39^ can be used to tune the system response timing with attosecond resolution.

It is worth noticing that we expect intra-band motion to be less important for Frenkel excitons like charge-transfer excitons in molecular crystals^8^ or interlayer excitons in layered solids^7^. Therefore, the sub-fs optical response of these excitons could qualitatively differ from what is reported here.

To conclude, we investigated ultrafast core-exciton dynamics around the L_2,3_ edge in MgF_2_ single crystal with ATRS. Simultaneous calibration measurements allowed us to perform a direct and unambiguous comparison with theoretical results, addressing the dual nature of the excitonic quasi-particle from a different perspective. In particular, we found that while the exciton dynamics unfolding on the first few femtoseconds originate from the optical Stark effect and can be understood invoking just the atomic character of the quasi-particle, the physical processes happening on an attosecond time scale during light–matter interaction (i.e. intra-band motion and DFKE) are typical of the condensed state of matter. Moreover, our theoretical simulations and analysis revealed that the atom-solid duality is general and exists for strongly bound excitons and also for delocalised valence excitons where the absolute timing of the system optical response can be controlled on attosecond time scale by tuning the exciton binding energy. Since these findings are not limited to the chosen target but hold, in general, for excitons originated by dispersive bands in solids, they set a new lever for the coherent control of excitonic properties in the close-to-petahertz regime.

## Methods

### Experimental setup

The setup used for the experiment reported in the main manuscript is described in detail in ref. ^23^. Single attosecond pulses (SAPs), centred around 42 eV photon energy, and 5 fs IR pulses (central wavelength 750 nm) are first focused onto a Ne gas target. The XUV pulses have a time duration of about 250 as while the IR peak intensity is set between 10^12^ and 10^13^ W/cm^2^. A time-of-flight (TOF) spectrometer records the photoelectron spectra as a function of the delay, *t*, between the IR and XUV pulses to perform an attosecond streaking experiment^40^. This allows us to retrieve the temporal characteristics of the two pulses and to obtain a precise calibration of the relative delay *t* by extracting the exact shape of the IR vector potential. A gold-plated toroidal mirror then focuses both beams onto the MgF_2_ crystal, where a thin gold layer deposited on a portion of the sample is used to calibrate the incident XUV photon flux and extract the energy-dependent sample reflectivity, *R*_*0*_*(E)*. For more details, see the Supplementary Notes 1–5.

### Data analysis

To study the different mechanisms underlying the transient features observed in Δ*R*/*R*, we decompose the pump-probe spectrogram in a slow and a fast component. To extract the slow component, we apply a low-pass frequency filter to the reflectivity spectrogram which is constant for frequencies below a cut-off frequency *fc* and decays with a super-Gaussian a profile $e^{\left( {\frac{{f - f_c}}{{2\sigma _f}}} \right)^n}$with coefficient *n* = 16 and width *σ*_*f*_ = 0.01 PHz. Since the fastest feature observed oscillates at twice the IR frequency 2*f*_IR_ ≈ 0.75 PHz, we decided to set *fc* to 1.5*f*_IR_ = 0.5621 PHz. Once the slow component has been extracted, the fast component of Δ*R*/*R* is simply obtained by subtracting the slow component from the total spectrogram. In the case of the experimental data, a high-frequency filter centred at 5*f*_IR_ = 1.8737 PHz is used to remove the fast noise from the data prior to slow and fast decomposition.

As discussed in the main manuscript, the femtosecond transient features of Δ*R*/*R* originate from the optical Stark effect (OSE) induced by the IR electric field. To extract the Stark shift ε from the experimental data, at each delay *t*, we fitted the sample reflectivity at the presence of the IR pump, *R*_IR_*(E, t)* with six Gaussian bells. Two Gaussians describe the background. Their parameters are derived from the static reflectivity *R*_*0*_*(E)*. The other four Gaussians are used to fit the bright and dark exciton features, doubled because of the Mg^2+^ 2*p* spin–orbit splitting. As observed for MgO^19^, the dark excitonic state is responsible for an increase of *R*_IR_*(E, t)* around *t* = *0* fs, which appears next to the bright excitonic peak, on the low-energy side, thus overlapping with the bright exciton signal which originates from *2p*_*3/2*_ state. Due to the energy overlap, it is not possible to fit accurately the contribution of the *2p*_*1/2*_-dark state transition as well as all the transitions involving the *2p*_*3/2*_ state. Therefore, we can obtain a reliable estimation of ε(*t*) only for the bright-exciton-*2p*_*1/2*_ transition which is found to follow the delay-dependent energy position of the maximum of Δ*R*/*R* around the A feature. The excitonic dipole *d(t)* is obtained by deconvoluting the delay-dependent Stark shift ε(*t*) with the envelope of the IR electric field^19^, directly extracted from the simultaneous streaking trace. The Auger decay rate γ and the phonon coupling ϕ can then be evaluated by modelling the excitonic dipole with the function ${\it{d}}({\it{t}})\sim e^{ - \gamma t}e^{\phi ({\it{t}})}$^18,19^. For more details see Supplementary Note 9.

The absolute phase delay between the fast transient feature of Δ*R*/*R* and the IR electric field is evaluated following the approach reported in^15,31^. First the IR vector potential is extracted from the simultaneous streaking trace by means of a 2D fitting procedure based on the analytical model reported in^41^. Then the phase difference between the transient features in the differential reflectivity and *E*_IR_^2^ is directly evaluated by multiplying the energy-dependent Fourier transform of the first with the complex conjugate of the Fourier transform of the latter. The product thus constructed automatically peaks at the common frequency between the signals and has a phase equal to their phase difference Δφ. As Δφ could be frequency-dependent, we evaluate the average value around 2*f*_IR_ using the local intensity of the Fourier transform product as weight. The standard deviation is obtained calculating the second momentum of this distribution. Finally, the phase delay τ is given by the ratio between Δφ and the beating frequency 2*f*_IR_. The main results reported in Fig. 3c represent an average over four independent transient reflection measurements conducted under similar conditions and weighted by the inverse of their individual experimental uncertainty. The final error accounts both for the mean measurement error and for the statistical deviation between the independent measurements. For further details, see Supplementary Note 7. We note that τ has the opposite sign of the pump-probe delay modulations (compare the black curves in Fig. 3a, c). This originates from the fact that a positive pump-probe delay *t* means that the IR pulse is coming later (IR behaving as a probe), but for the sake of an easier interpretation, we chose a positive τ to mean that the system has a delayed response with respect to the IR field in real time (IR behaving as a pump).

### Theoretical model

To investigate the microscopic mechanism of the experimental observation, we describe the dynamical system under the intense IR pulse and the weak XUV pulse based on the following ansatz:

1 $$\left| {{\Psi}\left( t \right)} \right\rangle = \left. {\left| {{\mathrm{{\Phi}}}_{{\mathrm{GS}}}} \right.} \right\rangle + \mathop {\sum }\limits_k c_k\left( {\it{t}} \right)\hat a_{c,k + eA_{{\mathrm{IR}}}\left( t \right)/\hbar c}^\dagger \hat a_{v,k + eA_{{\mathrm{IR}}}\left( {\it{t}} \right)/\hbar c}\left| {{\Phi}_{{\mathrm{GS}}}} \right\rangle ,$$

where $\left| \right.\Psi\left(t\right) \left\rangle\right.$ is the wave-function of the dynamical system, $\left| \right.{{\mathrm{{\Phi}}}_{{\mathrm{GS}}}} \left\rangle\right.$ is the ground state wave-function of the matter, $\hat {a}_{c,k}^\dagger \left( \hat {a}_{v,k} \right)$ is a creation (annihilation) operator for the conduction (valence) state at the Bloch wavenumber *k*, and *c*_*k*_(*t*) is an expansion coefficient. Here, the vector potential of the IR field is denoted as *A*_IR_(*t*). Note that the ansatz in Eq. (1) is a linear combination of single-particle single-hole states and is in line with the Tamm-Dancoff approximation. Treating the excitation from the ground state to electron–hole states by the XUV field perturbatively, the equation of motion for the coefficient *c*_*k*_(*t*) is given by

2 $$i\hbar \dot c_k\left( {\it{t}} \right) = \left[ {\mathop {\sum }\limits_{k^\prime } {\it{H}}_{kk^\prime }\left( {\it{t}} \right){\it{c}}_{k^\prime }\left( {\it{t}} \right)} \right] + {\it{E}}_{{\mathrm{XUV}}}\left( {\it{t}} \right) \cdot {\it{D}}_{k + eA_{{\mathrm{IR}}}\left( t \right)/\hbar c},$$

where *E*_XUV_ (*t*) is the electric field of the XUV pulse, *D*_*k*_ is the transition dipole moment between the ground state and the electron–hole state at the Bloch wavevector *k*, and *H*_*kk*′_ (*t*) is the electron–hole Hamiltonian. We employ the Wannier-Mott model^26,27^, and the electron–hole Hamiltonian is given by

3 $${\it{H}}_{kk^\prime }\left( {\it{t}} \right) = \delta _{kk^\prime }\left[ {{\it{E}}_{\it{g}} + \frac{1}{{2\mu }}\left( {\hbar k + \frac{e}{c}{\it{A}}_{{\mathrm{IR}}}\left( {\it{t}} \right)} \right)^2} \right] - {\it{V}}_{kk^\prime }$$

where *E*_g_ is the direct gap of the matter, *μ* is the effective electron–hole mass, and *V*_*kk*′_ is the Coulomb interaction, which we model with the one-dimensional soft Coulomb interaction. Note that, by diagonalizing *H*_*kk*′_ (*t* = 0), one obtains exciton states as bound states of electrons and holes as shown in Fig. 1c.

We solve the Schrödinger equation, Eq. (2), with an open-source code^42^ and evaluate physical observables with the time-evolving wave-function $\left| {{\Psi}\left( {\it{t}} \right)} \right\rangle$, For example, the induced electric current density can be computed as $J_{{\mathrm{XUV}}}\left( t \right) = \left\langle {{\Psi}\left( {\it{t}} \right)\left| {\hat J} \right|{\Psi}\left( {\it{t}} \right)} \right\rangle /{\Omega}$, where *Ĵ* is the current operator and Ω is the crystal volume. Furthermore, the linear susceptibility *χ*_exc_ (*ω*) can be evaluated as

4 $$\chi _{{\mathrm{exc}}}\left( \omega \right) = i\frac{{\sigma _{{\mathrm{exc}}}\left( \omega \right)}}{\omega } = \frac{i}{\omega }\frac{{\tilde J_{{\mathrm{XUV}}}\left( \omega \right)}}{{\tilde E_{{\mathrm{XUV}}}\left( \omega \right)}},$$

where $\sigma _{{\mathrm{exc}}}\left( \omega \right)$ is the optical conductivity evaluated as the ratio of the current and the electric field in the frequency domain. We model the dielectric function of MgF_2_ by combining the core-exciton susceptibility *χ*_exc_ (*ω*) and the valence contribution as

5 $$\epsilon_{{\mathrm{MgF}}_2}\left( \omega \right) = {{\epsilon }}_{{\mathrm{valence}}}\left( \omega \right) + 4\pi c\left[ {\chi _{{\mathrm{exc}}}\left( \omega \right) + \frac{1}{3}\chi _{{\mathrm{exc}}}\left( {\omega + {\Delta}_{{\mathrm{SO}}}} \right)} \right],$$

where ${\it{\epsilon }}_{{\mathrm{valence}}}\left( \omega \right)$ is the valence contribution, *c* is a fitting parameter and Δ_SO_ is the spin–orbit split. By optimising ${\it{\epsilon }}_{{\mathrm{valence}}}\left( \omega \right)$ and *c*, the dielectric function and the reflectivity of MgF_2_ can be well reproduced by the above model (see Fig. 1b).

With the final goal being to investigate the transient reflectivity of the XUV pulse under the presence of the IR pulse, we compute the electron dynamics by solving Eq. (2) with both the IR and XUV fields. Then, we further compute the current with the time-dependent wave-function $\left. {|{\mathrm{{\Psi}}}\left( t \right)} \right\rangle$. Following the above procedure, Eqs. (4) and (5), the transient dielectric function under the presence of the IR field and the corresponding transient reflectivity can be evaluated. The computed transient reflectivity by the Wannier-Mott model is shown in Fig. 1e.

In order to obtain further insight into the phenomena, we construct two idealised models. One is the pure-exciton model, and it is designed to exclude the crystalline nature of the dynamics. The other is the pure-crystal model, and it is designed to exclude the atomic nature. The pure-exciton model is a three-level model constructed by the following three states; the ground state $\left. {|{\Phi}_{{\it{{\mathrm{GS}}}}}} \right\rangle$, the bright exciton state and the dark exciton state (see Fig. 1c). Hence the pure-exciton model allows us to extract the nature of the discrete energy levels (atomic nature). Note that the pure-exciton model is constructed by the subspace of the above Wannier-Mott model because the bright exciton state is the ground state of *H*_*kk*′_ (*t* = 0) and the dark exciton state is the first excited state. The pure-crystal model, instead, consists of the conduction band (CB) but excludes the exciton states (see Fig. 1c). Such a model can be realised by setting the electron–hole attraction *V*_*kk*′_ to zero, and it is identical to the parabolic two-band model used to discuss the dynamical Franz-Keldysh effect^30^. Therefore, the pure-crystal model allows us to extract the contribution purely from the intra-band motion (crystal nature) accelerated by the IR field. For a detailed discussion of the theoretical model^42^, see Supplementary Notes 10–19.

## Supplementary information

Supplementary Information

## Data Availability

All data generated and analysed during this study are available from the corresponding author upon reasonable request.

## Code availability

All the custom codes used in this study are available from the corresponding author upon reasonable request.
