# Supplementary material for: Unravelling the intertwined atomic and bulk nature of localised excitons by attosecond spectroscopy
Source: Nat Commun. 2021 Feb 15;12:1021. doi: 10.1038/s41467-021-21345-7 (PMC7884782; doi:10.1038/s41467-021-21345-7)
Supplement: Supplementary file 1 — Supplementary Information [file 41467_2021_21345_MOESM1_ESM.pdf]

Supplementary information for: Unravelling the intertwined atomic and bulk nature of localised excitons by attosecond spectroscopy

Matteo Lucchini[1,2*], Shunsuke A. Sato[3,4], Giacinto D. Lucarelli[1,2], Bruno Moio[1,2], Giacomo Inzani[1], Rocío Borrego-Varillas[2], Fabio Frassetto[5], Luca Poletto[5], Hannes Hübener[4], Umberto De Giovannini[4,6], Angel Rubio[4,6], Mauro Nisoli[1,2]

[1]Department of Physics, Politecnico di Milano, 20133 Milano, Italy
[2]Institute for Photonics and Nanotechnologies, IFN-CNR, 20133 Milano, Italy
[3]Center for Computational Sciences, University of Tsukuba, Tsukuba 305-8577, Japan
[4]Max Planck Institute for the Structure and Dynamics of Matter, 22761 Hamburg, Germany
[5]Institute for Photonics and Nanotechnologies, IFN-CNR, 35131 Padova, Italy
[6]Nano-Bio Spectroscopy Group, Universidad del País Vasco, 20018 San Sebastian, Spain

*To whom correspondence should be addressed; E-mail: matteo.lucchini@polimi.it.

Materials and Methods

1 Experimental apparatus

The setup used for the experiment reported in the main manuscript is described in detail in Ref. [1]. Infrared (IR) pulses with a duration of 25 fs, central wavelength of 800 nm, repetition rate of 10 kHz and energy of 2 mJ are compressed with a hollow-core fiber setup [2] to 6 fs. The compressed pulses follow an evacuated beamline where they are first divided by a 70-30 beam splitter. The more intense part is focalized onto a gas cell, filled with Ar, to generate extreme-ultraviolet (XUV) attosecond pulses via high-order harmonic generation process. Since the

laser system is CEP-stabilized (shot-to-shot standard deviation of 320 mrad [3]), it is possible to apply the ionization gating technique [4] to generate single attosecond pulses (SAPs, hereafter probe) centered around 42 eV and with a spectrum extending over the Mg $L_{2,3}$ edge, located around 56 eV [5, 6]. The weaker part of the IR beam (hereafter pump), follows a separate path where the delay is controlled with a translation stage. After adjustment of the beam divergence, the pump beam is collinearly recombined with the probe by means of a drilled mirror. An Al metallic filter placed on the probe path, before the drilled mirror, allows efficient removal of the residual IR radiation after SAP generation. Both beams are then focused by a gold-plated toroidal mirror onto a gas target, located in the focal spot of a time-of-flight (TOF) spectrometer. A second toroidal mirror refocuses both beams onto the MgF_2 single crystal (see Sec. 2) placed into a reflectometer. Precise alignment of the sample in the focal position is achieved through a motorized sample holder which allows translation in the three spatial directions and rotation around the vertical axis [1]. The reflected part of the XUV radiation is finally collected by a gold-plated mirror and sent to an XUV spectrometer at the end of the line to measure the sample reflectivity (see Secs. 4 and 5). Due to the sequential double-foci geometry of our beamline, we can perform at the same time a photoelectron experiment in the first focus and a transient reflection experiment in the second. In this way, we can retrieve the temporal characteristics of pump and probe pulses with an attosecond streaking experiment [7, 8] and set a precise reference for the zero of the pump-probe delay axis of the simultaneous attosecond transient reflectivity experiment [1]. Thanks to this unique feature, we can access the energy-dependent attosecond phase delay of the observed transient features in the sample optical response with respect to the IR instantaneous pump field (see Sec. 7).

2 Sample description

The sample used for the experiment is a commercially available single crystal of magnesium fluoride (MgF_2), from SurfaceNet GmbH. The sample is 6-mm wide, 12-mm high and has a thickness of 1 mm. The single crystal has a rutile-type structure oriented with the front face corresponding to the (001) crystal plane, whereas the edge orientation in the plane of incidence is identified by the crystal direction [010], as depicted in Fig. 1.

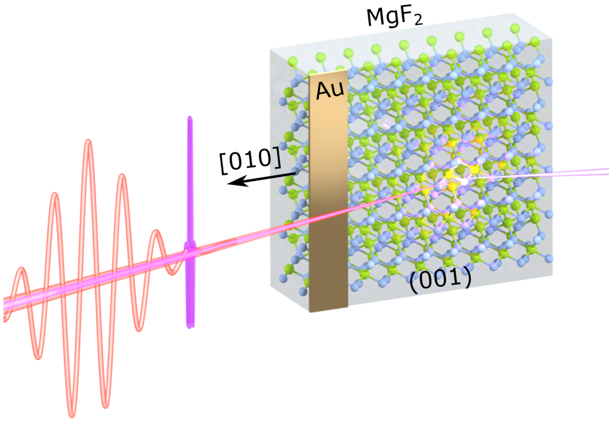

Figure 1: Schematic of the MgF_2 sample used in the experiment. The size of the sample is $12 \times 6 \times 1$ mm^3. The front face is parallel to the (001) crystal plane, whereas the edge orientation in the plane of incidence is aligned to the [010] crystal direction. The gold layer is 2-mm wide and has a thickness of 50 nm, deposited by vapour deposition.

The front side is treated with a top-grade chemo-mechanical polishing procedure, typically used for epitaxial application, with a residual surface roughness that is nominally better than the lattice constant.

Before running the experiment, we deposited a layer of gold on one side of the front face of the sample, to unlock the possibility to measure reference spectra, useful for static experiments, minimizing the sample holder movements. This reduces the uncertainty in the alignment of the optical elements and prevents any artifact that may arise in the measurement of the static reflectance (Sec. 4). The Au layer is 2 mm in width, 50-nm thick and was deposited by vapour

deposition in thermal mode. With this procedure, a high-purity amount of gold is melted in a ceramic heater and a metal vapour forms. Since the procedure is done in high vacuum, the vaporized gold particles have a long mean free path and travel straight to the target, where they condense. By means of a mask, gold deposition is limited to a precise region. The thickness is controlled by measuring the tiny variations in the weight of the sample and controlling the speed of evaporation.

3 Evaluation of MgF_2 critical angle

To choose the incidence angle for the experiment (called critical angle), we followed the approach of Kaplan and coworkers [9, 10]. We define $\tilde{n}$ as the complex refractive index of MgF_2:

$$\tilde{n} = n + ik \tag{1}$$

where i is the imaginary unit and n and k are, respectively, the real and the imaginary part of the complex refractive index. The reflectivity for s-polarized incident radiation can be computed using the Fresnel equations:

$$R_s = |r_s|^2 = \left| \frac{\cos(\theta) - \sqrt{\tilde{n}^2 - \sin^2(\theta)}}{\cos(\theta) + \sqrt{\tilde{n}^2 - \sin^2(\theta)}} \right|^2 \tag{2}$$

where r_s is the reflection coefficient for s-polarized light and θ is the incidence angle with respect to the surface normal. The reflectivity in the photon energy range between 25 and 65 eV and for an incidence angle θ between 60° and 85° is shown in Fig. 2(a). The values for $\tilde{n}$ have been taken from Hanson *et al.* [6]. A strong variation in the reflectivity can be observed around ~ 54.6 eV, where the excitonic feature is present, independently from the value of the incidence angle θ.

By definition, the sample dielectric constant ε can be written as the sum of its real and imaginary part:

$$\varepsilon = \tilde{n}^2 = \varepsilon_1 + i\varepsilon_2 \tag{3}$$

The derivative of the reflectivity with respect to ε_1 and ε_2 thus become:

$$\frac{\partial R_s}{\partial \varepsilon_1} = 2\Re\left\{r_s^* \cdot \frac{\partial r_s}{\partial \varepsilon_1}\right\}, \quad \frac{\partial R_s}{\partial \varepsilon_2} = 2\Re\left\{r_s^* \cdot \frac{\partial r_s}{\partial \varepsilon_2}\right\} = -2\Im\left\{r_s^* \cdot \frac{\partial r_s}{\partial \varepsilon_1}\right\} \tag{4}$$

Furthermore, following the definition of r_s given in Eq. (2):

$$\frac{\partial r_s}{\partial \varepsilon_1} = -\frac{\cos(\theta)}{\sqrt{\tilde{n}^2 - \sin^2(\theta)} \cdot \left[\cos(\theta) + \sqrt{\tilde{n}^2 - \sin^2(\theta)}\right]^2} = -i\frac{\partial r_s}{\partial \varepsilon_2}. \tag{5}$$

Therefore we can define the sensitivity of the reflectivity to the real and imaginary part of the dielectric constant as:

$$S_{\varepsilon_1} = \frac{\varepsilon_1}{R_s} \cdot \frac{\partial R_s}{\partial \varepsilon_1}, \quad S_{\varepsilon_2} = \frac{\varepsilon_2}{R_s} \cdot \frac{\partial R_s}{\partial \varepsilon_2}. \tag{6}$$

The values of S_{ε_1} and S_{ε_2} computed in the same energy-angle space as before are reported in Figs. 2(b) and 2(c). Also in this case, for any incidence angle, we observe strong variations at ~ 54.6 eV. For those points where $S_{\varepsilon_2} = 0$, marked by the dashed curves in Figs. 2(c) and 2(d), the dependence of the reflectivity on the imaginary part of the complex dielectric function of MgF_2 is minimized.

An alternative approach consists in considering the total external reflection. Since the real part of the complex refractive index of MgF_2 is smaller than one in the XUV spectral region, it is possible to define the critical angle for total external reflection [11] as:

$$\theta_c = \arcsin(n). \tag{7}$$

At the critical angle, the radiation is completely reflected from the surface and its absorption is minimized. The critical angle, reported in Fig. 2(d) as a solid line, closely tracks the zeros of the sensitivity to the imaginary dielectric function ε_2 in the region of the excitonic feature.

Summarizing the above findings, we chose an incidence angle of $73.5°$ to perform the transient reflection experiments reported in the main manuscript. This value is equal to the critical angle at the energy of the excitonic feature (~ 54.6 eV). Moreover, at this angle the sensitivity

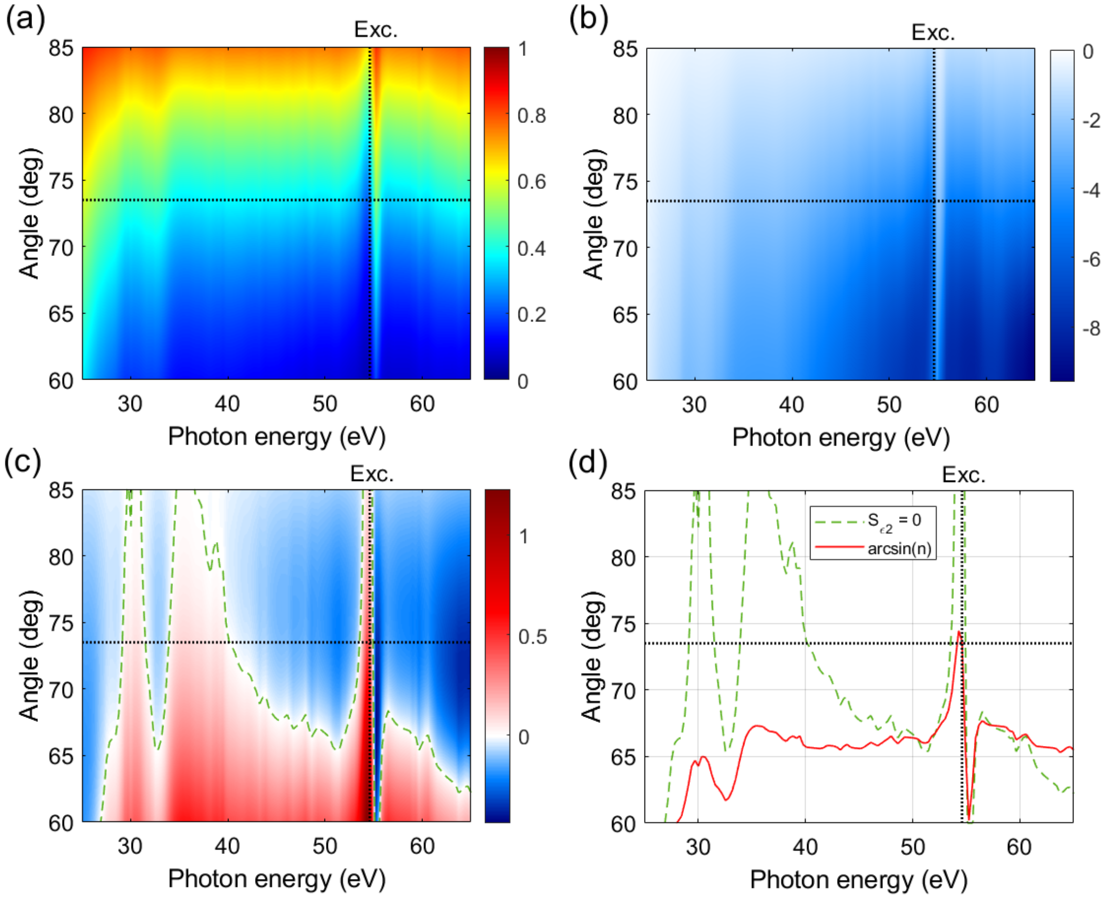

Figure 2: **(a)** Reflectivity of MgF_2 for s-polarized light. **(b)** Sensitivity S_{ε_1} to the real part of the dielectric constant. **(c)** Sensitivity S_{ε_2} to the imaginary part of the dielectric constant of MgF_2. The green dashed curve marks the points where the sensitivity is zero. **(d)** Incidence angle at which $S_{\varepsilon_2} = 0$ as a function of photon energy (green dashed curve), compared to the critical angle for total external reflection (red solid curve). In all panels the black dotted horizontal line identifies the experimental incidence angle (73.5°).

to the imaginary part of the dielectric function is close to zero and it is one order of magnitude smaller than the sensitivity to the real part of the dielectric function, thus assuring an easier interpretation of the reflectivity data. It is worthy to stress that at the critical angle the static reflectivity still depends on the imaginary part of the dielectric function (refractive index). As the reflectivity is more sensitive to variations of $\Re\{n\}$ around 1, a dispersive profile in $\Re\{n\}$ translates into a less dispersive and more peak-like structure for R_s, in agreement with what reported by Hanson et al. [6].

4 Static reflectivity measurements

The static reflectivity $R_0(E)$ of MgF_2 as a function of the XUV photon energy E can be determined by comparing the same XUV photon spectrum as reflected by the sample and by a known reference material. In this experiment, we choose as a reference material a 50-nm gold layer directly deposited on the sample, as described in Sec. 2.

If $I(E)$ indicates the intensity of the incoming XUV radiation, $R_0(E)$ the reflectivity of the MgF_2 sample and $R_M(E)$ the reflectivity of the folding gold mirror used to send the beam into the spectrometer, upon reflection on the sample the SAP spectrum measured at the end of the line will be equal to:

$$I_{MgF_2}(E) = R_M(E)\, R_0(E)\, I(E). \tag{8}$$

Moving the gold layered part of the sample onto the beam path, we will instead measure an intensity given by:

$$I_{Au}(E) = R_M(E)\, R_{Au}(E)\, I(E), \tag{9}$$

where $R_{Au}(E)$ is the reflectivity of the gold layer deposited directly onto the sample. From the two previous expressions, it is therefore possible to derive the reflectivity $R_0(E)$ of MgF_2 as:

$$R_0(E) = \frac{I_{MgF_2}(E)}{I_{Au}(E)} \cdot R_{Au}(E). \tag{10}$$

Since the reflectivity $R_{Au}(E)$ of gold can be computed from Eq. (2) starting from the known optical constants of gold [12], Eq. (10) shows that it is possible to determine $R_0(E)$ by measuring the same SAP spectrum reflected first by the sample I_{MgF_2} and then by the gold layer I_{Au}.

To minimize the error stemming from any experimental instability, the measurement is performed as follows. At first we measure the spectrum $I_{Au,1}(E)$ after reflection onto the gold layer. The sample is then placed in position to measure $I_{MgF_2}(E)$. The sample is finally moved in the initial position in order to measure a second spectrum $I_{Au,2}(E)$. The total reference spectrum is computed as the average between the two acquired spectra as reflected by the gold layer:

$$I_{Au}(E) = \frac{I_{Au,1}(E) + I_{Au,2}(E)}{2}, \tag{11}$$

and used to obtain $R_0(E)$ through Eq. (10).

To acquire a spectrum, the acquisition system collects 100 independent measurements, with an integration time of 100 ms each. The final spectrum is given by the average of all independent acquisitions, while the measurement uncertainty can be estimated with the standard deviation:

$$\sigma_X = \sqrt{\frac{\sum_{i=1}^{N}(x_i - \bar{x})^2}{N-1}} \tag{12}$$

where in our case x_i is the single intensity measurement, $\bar{x}$ is the average of all the intensity measurements and N is the number of measurements (in this case, $N = 100$). The measured spectrum $I_{MgF_2}(E)$ after sample reflection and obtained following the procedure above is reported in Fig. 3(a) as a red solid curve. The red shaded area represents twice its standard deviation $\sigma_{MgF2}(E)$ obtained with Eq. (12).

For the reference spectrum I_{Au}, the total uncertainty is given by the sum of standard deviations the two independent measurements:

$$\sigma_{Au}(E) = \sqrt{\sigma^2_{Au,1}(E) + \sigma^2_{Au,2}(E)}. \tag{13}$$

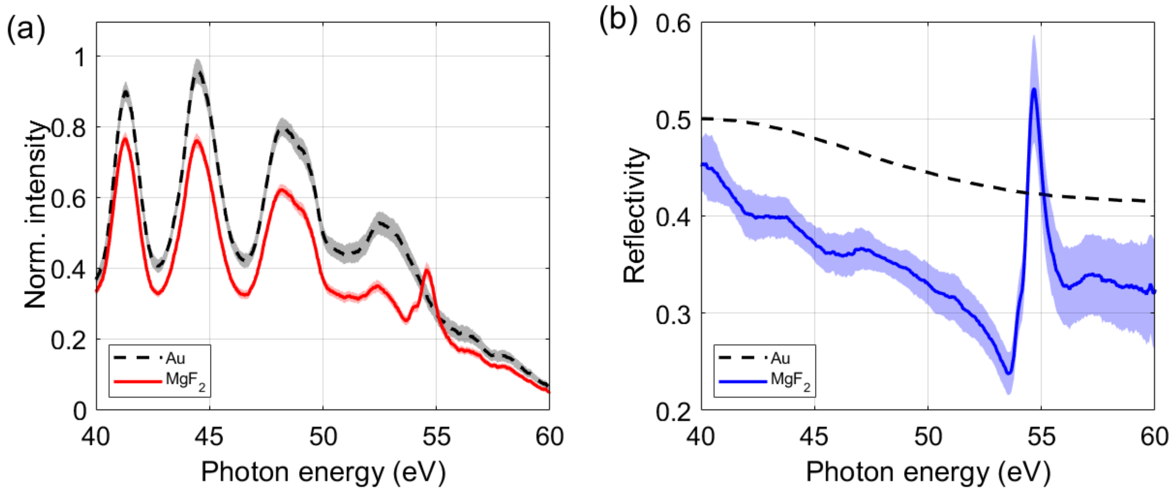

Figure 3: **(a)** Black dashed line: reference spectrum $I_{Au}(E)$ reflected by the gold layer deposited directly onto the MgF_2 sample. Red solid line: intensity spectrum $I_{MgF_2}(E)$ reflected by the MgF_2 sample. Shaded areas represent twice the standard deviation over repeated measurements. **(b)** Black dashed line: theoretical reflectivity $R_{Au}(E)$ of gold [12]. Blue solid line: measured reflectivity $R_{MgF_2}(E)$ of the MgF_2 sample. The shaded area represents twice the standard deviation computed propagating the error.

An example of I_{Au} obtained with this procedure is shown by the black dashed curved and shaded area in Fig. 3(a).

As mentioned above, Eq. (10) can be directly used to compute the average experimental reflectivity of MgF_2 $R_0(E)$. Propagating the measurement errors, the associated standard deviation is finally given by:

$$\sigma_{R_0}(E) = |R_0(E)| \cdot \sqrt{\left(\frac{\sigma_{MgF_2}(E)}{I_{MgF_2}(E)}\right)^2 + \left(\frac{\sigma_{Au}(E)}{I_{Au}(E)}\right)^2}. \tag{14}$$

The $R_0(E)$ obtained starting from the spectra in Fig. 3(a) and evaluating the uncertainty with the above equation is shown in Fig. 3(b) by the blue curve and shaded area. The dashed black curve represents instead the $R_{Au}(E)$ adopted, as computed from the optical constants reported by Werner *et al.* [12]. A detailed discussion of the features observed in $R_0(E)$ is reported in the main manuscript.

5 Transient reflection measurements

The main experiment consists in the assessment of the effect of the IR pulse on the sample differential reflectivity $\Delta R/R$ defined as:

$$\frac{\Delta R(E,t)}{R(E)} = \frac{R_{IR}(E,t) - R_0(E)}{R_0(E)} \tag{15}$$

where E is the XUV photon energy, t is the delay between the IR and XUV pulses, $R_{IR}(E,t)$ is the XUV reflectance in the presence of the IR light and $R_0(E)$ is the static unperturbed reflectance (Sec. 4). As described in the previous section, the sample reflectivity is proportional to the measured XUV spectrum after reflection. Therefore, we can evaluate $\Delta R/R$ by computing

$$\frac{I_{IR}(E,t) - I_0(E)}{I_0(E)}, \tag{16}$$

where $I_{IR}(E,t)$ is the XUV spectrum measured at a precise pump-probe delay t when the IR pulse is on, $I_0(E)$ is the XUV spectrum without the pump pulse, which is measured by blocking the IR path with a mechanical shutter [1]. The shutter acts synchronously with the rest of the acquisition system, to make sure that the signals with IR on and off are correctly separated, and commutes with a frequency of about 1 Hz. In a typical measurement, we collect 120 spectra with and 120 without the IR pulses per delay, each one measured with an integration time of 100 ms, corresponding to 1000 lasers shots. We span a delay range of 30 fs, in steps of 250 as, whereas the mean energy resolution in the vicinity of 45 eV is about 30 meV. With this approach, one can measure the transient reflectance without knowing the incoming XUV intensity. For each transient reflection scan, we acquire a simultaneous streaking trace [7] used to calibrate the pump-probe delay as described in the main manuscript and in Ref. [1]. Synchronization between photon and photoelectron acquisition at each delay is obtained through a software which controls the value of the pump-probe delay and moves to the next data point only when both acquisitions are done.

To ensure that different measurements are taken under the same experimental conditions, we adjust the IR pump parameters prior starting each acquisition. For such purpose, we measure the pump average power with a powermeter, while the spatial properties onto the target position are retrieved with a beam profiler. The temporal characteristics of the IR pulse can instead be measured with an attosecond streaking experiment [7] in the first focus of the beamline [1].

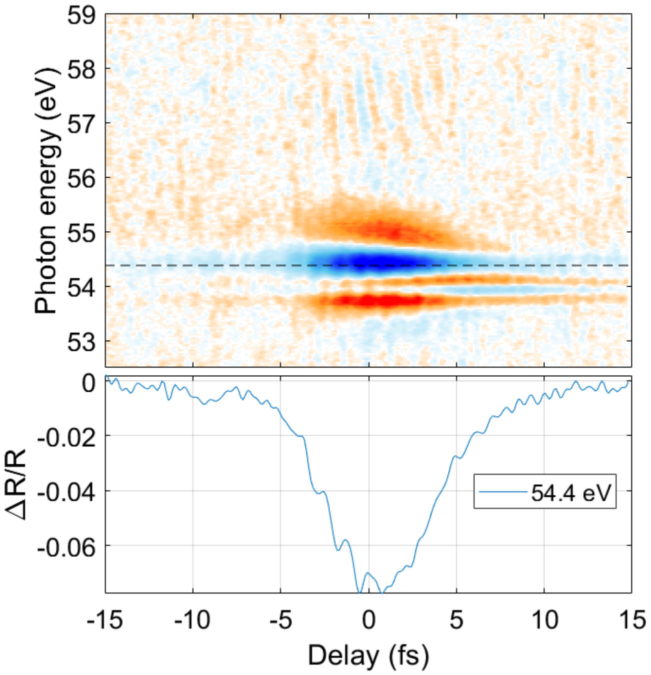

Figure 4: Upper panel: extended data from Fig. 1d of the main manuscript. Lower panel: differential reflectivity profile extracted at 54.4 eV, marked by the horizontal black dashed line in the top panel. The residual signal at negative delays goes to zero before -15 fs, becoming comparable with the measurement noise.

6 Imperfect IR pulse contrast

The non-zero signal observed at negative delays in Fig. 1d of the main manuscript comes from a non-perfect IR pulse contrast. We note that this signal, smaller than 4%, goes to zero for delays smaller than -15 fs (see Fig. 4) and does not influence the data analysis and conclusions of this

work.

7 Phase delay extraction

To extract the phase delay between the fast transient features in the sample differential reflectivity $\Delta R/R$ and the square of the IR pump electric field $E_{IR}^2(t)$, we first need to evaluate the IR vector potential from the simultaneous streaking trace. Since, at each delay, the photoelectron spectrum is streaked by a quantity proportional to the instantaneous IR vector potential, the easiest way to obtain $A_{IR}(t)$ is to evaluate the center of mass of the spectrogram [13]. Nevertheless, the presence of a satellite in the attosecond radiation and the measurement noise can alter the center of mass and cause a deviation from $A_{IR}(t)$ (see Fig. 5(a) and black curve in Fig. 5(c)). In order to minimize these effects we decided to follow a different approach and use a 2D fitting procedure based on an analytical model [14]. In this way, if needed, we can isolate the trace generated by the main attosecond pulse (Fig. 5(b)) and obtain a more reliable prediction for the center of mass (blue curve in Fig. 5(c)) and $A_{IR}(t)$ (Fig. 5(d))). We note that the fitting procedure assumes a perfect IR pulse contrast, cleaning any possible pedestal or prepulse. This has no influence on the phase extraction procedure.

To evaluate the energy dependent phase delay τ we take the transient reflectivity spectrogram $\Delta R/R$ measured simultaneously with the streaking trace (Fig. 6(a)) and perform a line-by-line Fourier transform (Figs. 6(b), 6(c)). Following the method used in Refs. [15, 13], τ can be directly evaluated by multiplying the Fourier transform of $\Delta R/R$ with the complex conjugate of the Fourier transform of the associated squared IR vector potential. The product of the two Fourier transforms, $P(E, \omega)$, indeed peaks at the common frequency (black dashed vertical lines in Figs. 6(b) and 6(c)) and its phase yields directly the phase difference between $\Delta R/R$ and $A_{IR}^2(t)$.

From the 2D phase of $P(E, \omega)$ we estimate the behavior of the phase difference $\varphi(E)$

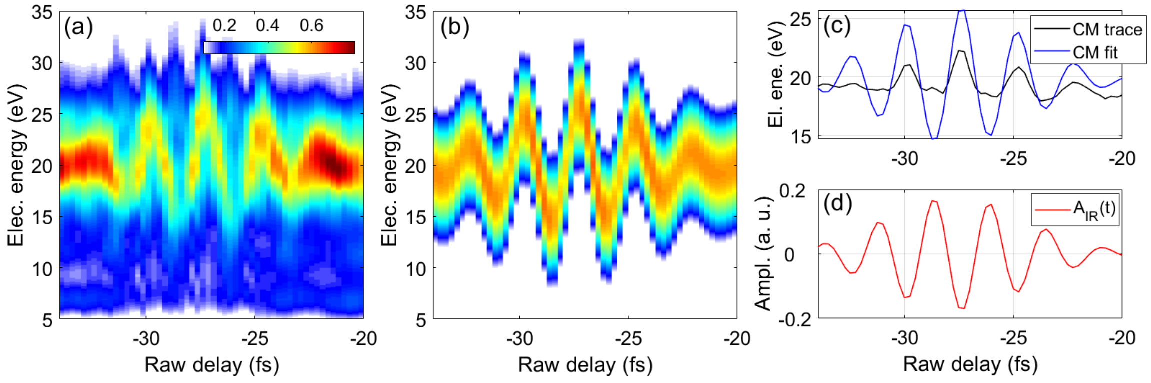

Figure 5: **(a)** Experimental streaking spectrogram characterized by the presence of a weak satellite pulse. **(b)** Result of the 2D fitting procedure, extracting the streaking trace associated only to the main attosecond pulse. **(c)** Comparison between the raw center of mass (black curve) and the center of mass extracted with the fitting procedure (blue curve). **(d)** IR vector potential $A_{IR}(t)$ as retrieved with the fitting procedure.

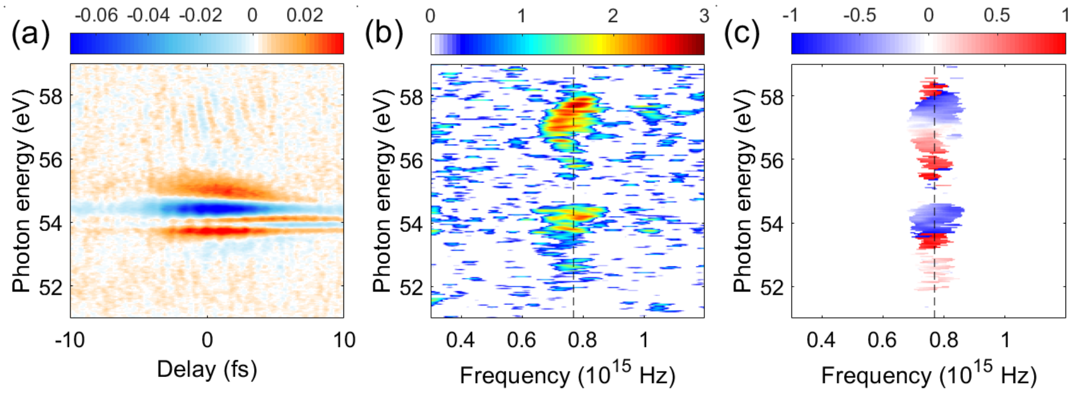

Figure 6: **(a)** Example of differential reflectivity spectrogram $\Delta R/R$. **(b)**, **(c)**, Spectral intensity and phase, respectively, of the line-by-line Fourier transform of the data in (a). In (b) the color scale represents the logarithm of the spectral intensity. In (c) the phase is plotted in units of π and only for those points which are intense enough to be above the noise level. In both panels the vertical black dashed line marks the common frequency with the IR pulse.

through a weighted average were the weight is represented by the spectral intensity of the product between the Fourier transforms (modulus square of $P(E, \omega)$):

$$\varphi(E) = \frac{\int |P(E,\omega)|^2 \, \mathrm{angle}\{P(E,\omega)\} \, d\omega}{\int |P(E,\omega)|^2 \, d\omega} \tag{17}$$

In order to reduce the effect of noise over the extraction of $\varphi(E)$, the integrals in the above equation run over the those Fourier frequencies, ω, for which $|P(E,\omega)|^2$ is higher than 50% of its maximum value.

In the same fashion, the uncertainty associated with the evaluation of $\varphi(E)$ can be estimated with the second momentum:

$$\sigma_\varphi^2(E) = \frac{\int |P(E,\omega)|^2 \left[\mathrm{angle}\{P(E,\omega)\} - \varphi(E)\right]^2 d\omega}{\int |P(E,\omega)|^2 \, d\omega} \tag{18}$$

The phase delay $\tau_i(E)$ can then be obtained dividing $\varphi(E)$ by $2\omega_{IR}$ and subtracting the propagation delay τ_{prop} measured by placing a second TOF spectrometer at the solid target position in order to perform a double-RABBITT measurement [1]. τ_{prop} accounts for any phase difference between the first and the second focus of the beamline, originating from alignment imperfections or unavoidable geometrical factors [16]. Indeed, an inhomogeneous delay across the sample surface can induce errors in the calibration of the absolute delay. As the Gouy phase changes its sign across the laser focus, its effect is minimized by the spatial average performed during detection. Finally, since the TOF used for the calibration experiment in the solid target region integrates over a focal region comparable to the interaction area on the solid sample, any asymmetric effect along the focus can also be compensated. It is worth noticing that our procedure does not account for a non-zero attosecond chirp, which may give an energy dependent propagation delay and therefore alter the absolute phase delay extracted on the solid sample. This effect is minimized choosing the thickness of the Al filters used to remove the residual IR light, in order to compensate the attochirp. A typical value of the measured attochirp for our pulses is of the order of $-0.3\,\mathrm{as}^2$, therefore any distortion due to a non-flat attosecond spectral

phase will be smaller than the experimental error bars on the extracted phase delay.

The total uncertainty of a single measurement is given by $\sigma^2_{\tau,i} = \sigma^2_{\varphi}(E)/2\omega^2_{IR} + \sigma^2_{prop}$. Figure 7 shows an example of phase delay τ_i superimposed to the associated transient reflectivity spectrogram. In order to directly compare τ_i with $\Delta R/R$, we set the pump-probe delay zero, $t = 0\,\mathrm{fs}$, to coincide with a zero of the IR vector potential $A_{ir}(t)$, or, equivalently, a maxiumum of the IR electric field $E_{IR}(t)$.

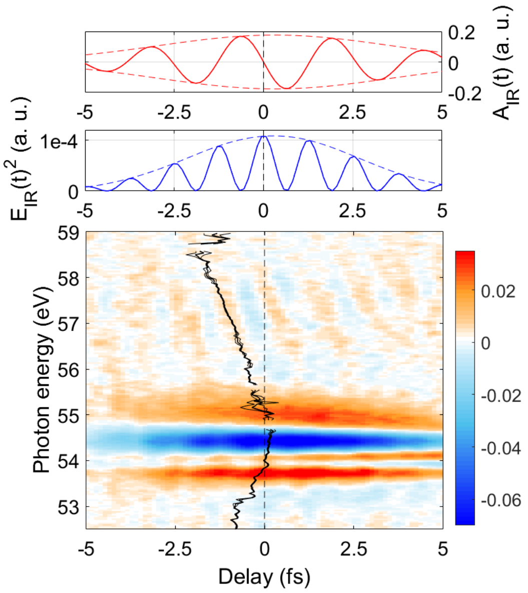

Figure 7: Upper panel, IR vector potential extracted with the 2D fit. Middle panel, associated electric field. Lower panel, comparison between the transient reflectivity trace (color plot) and the associated phase delay τ_i (black solid line), extracted following the procedure described in the text. The thinner black lines visualize twice the standard deviation.

The τ reported in Fig. 3c of the main manuscript is the result of an average over 4 independent transient reflection measurements conducted under similar conditions, and obtained by weighing each τ_i by the inverse of its uncertainty $\sigma_{\tau,i}$:

$$\tau = \frac{\sum \frac{\tau_i}{\sigma^2_{\tau,i}}}{\sum \frac{1}{\sigma^2_{\tau,i}}} \tag{19}$$

The associated error takes into account both for the mean measurement error and for the statis-

tical deviation between the independent measurements:

$$\sigma^2 = \sigma_\tau^2 + \delta\tau^2, \tag{20}$$

where:

$$\sigma_\tau = \frac{\sum \frac{1}{\sigma_{\tau,i}}}{\sum \frac{1}{\sigma_{\tau,i}^2}}, \qquad \delta\tau^2 = \frac{1}{N-1} \frac{\sum \frac{[\tau_i - \tau]^2}{\sigma_{\tau,i}^2}}{\sum \frac{1}{\sigma_{\tau,i}^2}}. \tag{21}$$

The results are reported in Fig. 8 (same as in Fig 4C of the main manuscript) where the region between 54.6 and 55.4 eV has been omitted because the associated oscillation amplitude in $\Delta R/R$ is too weak to justify a reliable delay extraction.

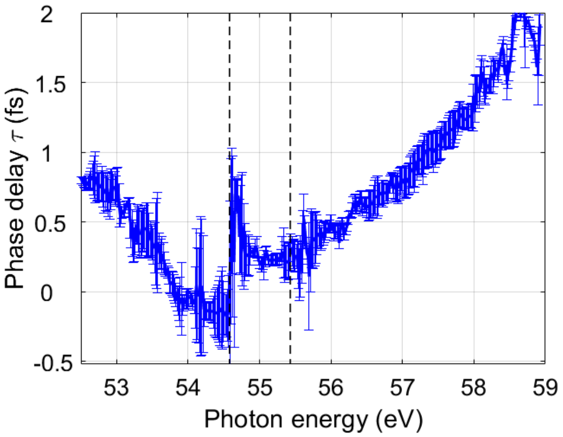

Figure 8: Phase delay τ obtained with Eq. (19). The error bars indicate the total uncertainty estimated with Eq. (20). The vertical black-dashed lines limit the energy region where the amplitude of the transient oscillation in $\Delta R/R$ is too weak to consider the extracted τ meaningful.

8 Decomposition in slow and fast component

To study the different mechanisms underlying the transient features observed in $\Delta R/R$, we decompose the pump-probe spectrogram in a slow and a fast component. To extract the slow component we first Fourier transform differential reflectivity spectrogram as done in Fig. 6(b). We then apply a low-pass frequency filter which is constant for frequencies below a cut-off

frequency f_c and decays with a supergaussian a profile $e^{\left(\frac{f-f_c}{2\sigma_f}\right)^n}$ with coefficient $n = 16$ and width $\sigma_f = 0.01\,$PHz. Since the fastest feature observed oscillates at twice the IR frequency $2f_{IR} \simeq 0.75\,$PHz, we decided to set f_c to $1.5f_{IR} = 0.5621\,$PHz. Finally we back-transform in order to return to the time domain. Once the slow component has been extracted, the fast component of $\Delta R/R$ is simply obtained by subtracting the slow component from the total spectrogram.

In the case of the experimental data, a high-frequency filter centered at $5f_{IR} = 1.8737\,$PHz is used to remove the fast noise from the data prior to slow and fast decomposition.

9 Stark-shift extraction and dipole deconvolution

As discussed in the main manuscript, the femtosecond transient features of $\Delta R/R$ originate from the optical Stark effect (OSE) induced by the IR electric field. To extract the Stark shift ε from the experimental data, at each delay t, we fitted the sample reflectivity at the presence of the IR pump, $R_{IR}(E,t)$ (Fig. 9) with six Gaussian bells. Two Gaussians describe the background. Their parameters are derived from the static reflectivity $R_0(E)$. The other four Gaussians are used to fit the bright and dark exciton features, doubled because of the Mg $2p$ spin-orbit splitting [17]. As discussed in the main text, the dark excitonic state is responsible for an increase of $R_{IR}(E,t)$ around $t = 0\,$fs (marked with B in Fig. 9), which appears next to the bright excitonic peak, on the low energy side [18], thus overlapping with the bright exciton signal which originates from $2p_{3/2}$ state. Due to the energy overlap, it is not possible to fit accurately the contribution of the $2p_{1/2}$-dark state transition as well as all the transitions involving the $2p_{3/2}$ state. Therefore we can obtain a reliable estimation of $\varepsilon(t)$ only for the bright-exciton - $2p_{1/2}$ transition (white curve in Fig. 9(a) and black curve with error bars in Fig. 9(b)), which is found to follow the delay-dependent energy position of the maximum of $\Delta R/R$ around the A feature (red curve in Fig. 9(b)). As the values extracted from the fitting procedure may

depend on the choice of the basis function used (Gaussians, Lorentzian, etc.), we decided to estimate $\varepsilon(t)$ with the shift of the $\Delta R/R$ maximum. We note that a more sophisticated 2D fitting procedure [19, 20, 18] could help to disentangle all the different excitonic contributions. Nevertheless, due to the complexity of the system under investigation and the short exciton life time, comparable to the IR pulse duration, the 2D-fitting model cannot be applied without an a-priori knowledge of the exciton-phonon coupling or exciton-Auger life time, necessary to reduce the number of active fitting parameter. Indeed, without an educated initial guess, the 2D fitting procedure can visually converge to the same $R_{IR}(E,t)$ with very different parameters. A detailed description of the exciton decay process, capable to accurately disentangle the different decaying mechanisms therefore requires specific modeling and additional measurements with shorter IR pulses, and hence goes beyond the scope of this work. Nevertheless, we can obtain a first estimation the exciton lifetime even without the need for complex fitting procedures.

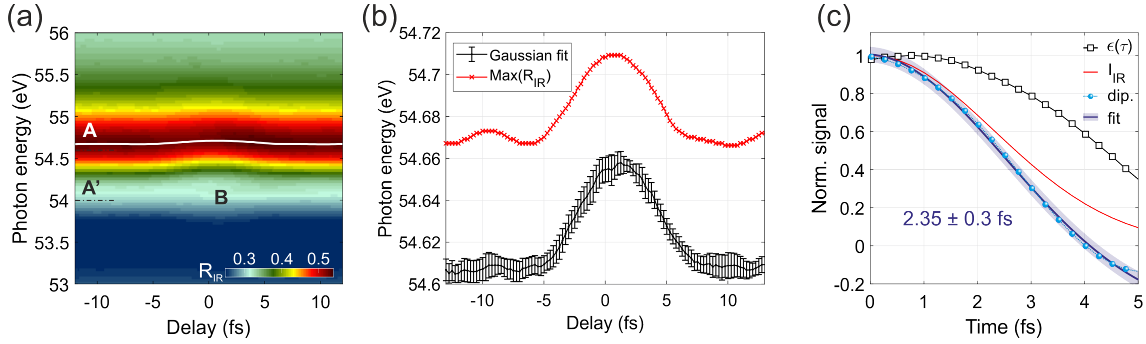

Figure 9: **(a)** Pumped sample reflectivity $R_{IR}(E,t)$ associated with the data in Fig. 2a of the main text. The white line tracks the reflectivity maximum around the excitonic feature A as a function of the delay, showing an evident Stark-shift in the region of pump-probe temporal overlap.**(b)** Delay-dependent optical Stark-shift $\varepsilon(t)$ extracted from (a). Black curve, $\varepsilon(t)$ of the bright exciton transition involving the Mg $2p_{1/2}$ state and extracted with the fitting procedure. The error bars represent the 95%-confidence interval of the fit. Red curve, maximum of the pumped reflectivity $R_{IR}(e,t)$ evaluated around the excitonic feature at 54.6 eV. **(c)** Dipole decay (light-blue dots) obtained by deconvolution of the experimental Stark-shift in (b) ($\varepsilon(t)$, black squares) with the experimental IR intensity profile (I_{IR}, red curve).The shaded blue area represents the 95% confidence interval of the fitting.

We concentrate only on the main bright exciton feature coming from the Mg $2p_{1/2}$ state and extract the excitonic dipole $d(t)$ by deconvoluting the delay-dependent Stark shift $\varepsilon(t)$ with the envelope of the IR electric field [18], directly extracted from the simultaneous streaking trace, as described in Sec. 7. The results are reported in Fig. 9(c).

According to the previous works by Moulet *et al.* [19] and by Mahan [21], in order to extract the Auger decay time and the phonon coupling to the excited state, it is possible to model the temporal evolution of the dipole moment $d(t)$ as follows:

$$d(t) \propto e^{-\gamma t} e^{-\phi(t)} \tag{22}$$

where γ is the Auger decay rate and the complex term $\phi(t)$ represent the effect of the phonons, in the following form

$$\phi(t) = \frac{M_0^2}{\omega_0^2}[(2N+1)(1 - cos\,\omega_0 t) - i(\omega_0 t - sin\,\omega_0 t)] \tag{23}$$

with N the phonon population, ω_0 the phonon frequency and M_0 the phonon coupling. If we limit our analysis to the real part of $\phi(t)$, thus considering only the effect of the phonons on the modulus of the dipole moment $d(t)$ and therefore only on the population, the above-mentioned expression can be simplified. If we now consider the phonon dynamics in the limiting case of $\omega_0 t \ll 1$, corresponding to a period of the phonons much longer than the Auger decay time $t_a = \frac{1}{\gamma}$, Eq. (23) can be simplified further as follows:

$$\phi(t) \approx \frac{M_0^2(2N+1)}{2} t^2 \tag{24}$$

Therefore we can fit $d(t)$ with a function $f(t) = Ae^{-t/t_a} e^{-M^2 t^2}$ where t_a denotes the Auger decay time and the positive term M, that we define such that $M^2 = \frac{M_0^2(2N+1)}{2}$, represents the effective coupling with the phonons. The former probably originates from an $L_{2,3}$(exciton)-VV decay process where the $2p$ Mg hole recombines with a valence electron, transferring energy to another valence electron which is further excited [22]. For the dipole reported in Fig. 9(c),

we found $t_a = 2.35 \pm 0.3\,\text{fs}$ and $M^2 = 0.072 \pm 0.011\,\text{fs}^2$. Therefore, the exciton decays within few femtoseconds, in agreement with what reported in literature for other core excitons in insulators [19, 18]. It is important to stress that regardless of the exact decaying mechanism, on a first approximation the slow component of $\Delta R/R$ can be explained with the same three-level system which describes a non-isolated atom whose temporal decay is partially influenced by the interaction with the environment (phonons). No dispersive states or band structure are necessary, on contrary to what observed for the attosecond dynamics.

Theoretical methods

To investigate the microscopic mechanism of the observed transient reflection signal, we employ quantum electron dynamics simulation based on the Wannier-Mott model [23, 24, 25]. The parameters of the model are determined by the *ab-initio* density functional calculation, or by fitting the experimental static reflectivity (see Sec. 4). Then, we simulate the pump-probe experiments and evaluate the transient reflection signal. As demonstrated in the main text, the model shows an excellent performance in comparison with the experimental results. Based on this fact, we further analyze the simulation results in order to obtain a microscopic insight into the light-induced exciton dynamics. In the following we will describe the model used in details.

10 Wannier-Mott model and electron dynamics under laser fields

Here, we first briefly describe the Wannier-Mott model [23, 24, 25]. Then, we further explain the theoretical approach used to compute the electron dynamics under IR and XUV laser fields.

In the Wannier-Mott model, electronic excited states of a semiconductor are described by a linear combination of electron-hole states,

$$|\Psi\rangle = \sum_{vck} c_{vc,k} \hat{a}^{\dagger}_{c,k} \hat{a}_{v,k} |\Phi_{gs}\rangle, \tag{25}$$

where v (c) denotes the valence (conduction) band index, $|\Phi_{gs}\rangle$ is the ground state wavefunction of the semiconductor, $\hat{a}_{v,k}$ is the annihilation operator for the valence state v at the Bloch wavevector k, and $\hat{a}^{\dagger}_{c,k}$ is the corresponding creation operator for the conduction band c. Note that this ansatz corresponds to the Tamm-Dancoff approximation for electronic excited states.

In the Wannier-Mott model, the electron-hole states are simply approximated by the single parabolic band dispersion, and the corresponding electron-hole Hamiltonian is given by

$$H_{k,k'} = \delta_{k,k'} \left[E_g + \frac{\hbar^2 k^2}{2\mu} \right] - V_{k,k'}, \tag{26}$$

where E_g is the electron-hole gap (direct gap), μ is the reduced electron-hole mass, and $V_{k,k'}$ is the interaction between electron-hole pairs. For simplicity, we consider the one-dimensional model in this work. The one-dimensional approximation has been widely employed to investigate the exciton dynamics, the attosecond electron dynamics as well as the strong field physics [26, 27, 19, 28]. We note that the physical mechanisms discussed in this work, the optical Stark effect (OSE) and the dynamical Franz-Keldysh effect (DFKE), are not very sensitive to the dimensionality of the model: the OSE can be described by a few-level system without the explicit treatment of the dimensionality, and the DFKE shows the same qualitative behavior in one, two and three-dimensional systems (see Fig. 13 and Refs. [29, 30]). Furthermore, the excellent agreement between the theoretical and experimental results reported in the main text clearly indicates the validity of the one-dimensional approximation.

For the electron-hole interaction, we employ the soft-Coulomb interaction as

$$V_{k,k'} = \frac{V_0}{\Omega} \int dx \frac{e^{i(k-k')\cdot x}}{\sqrt{x^2+1}} = V_0 \frac{\Delta k}{2\pi} 2K_0(|k-k'|), \tag{27}$$

where V_0 is the strength of the electron-hole interaction, Ω is the crystal volume, $K_0(x)$ is the modified Bessel function of the second kind. For the numerical simulation, the Bloch wavevector k is discretized by uniform grids with the spacing Δk. In this work, the spacing Δk is set to

$2 \times 10^{-3} a_0^{-1}$, and the wavevector k is truncated with the maximum wavevector k_{max} as

$$-k_{max} \leq k \leq k_{max}. \tag{28}$$

The maximum wavevector is determined by the maximum electron-hole energy, $E_{max} = \hbar^2 k_{max}^2 / 2\mu$, and we set E_{max} to 15 eV.

As described above, the theoretical model consists of a number of parameters. In this work, we set the reduced electron-hole mass μ to $0.24 m_e$ according to the result of the *ab-initio* density functional calculation with Becke-Johnson meta-GGA functional [31]. The interaction strength V_0 is set to $V_0 = 0.187$ a.u. so that the exciton binding reproduces the previously reported experimental value, 1.4 eV [5]. The electron-hole gap E_g is set to 55.8 eV to reproduce the exciton peak position in the static reflection (see Sec. 3). The Hamiltonian of the Wannier-Mott model is now given by Eq. (26). By diagonalizing the Hamiltonian, the excitonic states can be obtained as bound states of electron-hole pairs. The ground (bright) and the first excited (dark) excitonic states are evaluated with the above parameterization, and the computed energy levels are described in Fig. 1c in the main text.

Next, we describe the theoretical scheme to compute the laser-induced electron dynamics with the Wannier-Mott model. For this purpose, we extend the Hamiltonian of Eq. (26) by including laser fields as

$$H_{k,k'}(t) = \delta_{k,k'} \left[E_g + \frac{1}{2\mu} \left(\hbar k + \frac{e}{c} A_{IR}(t) \right)^2 \right] - V_{k,k'}, \tag{29}$$

where $A_{IR}(t)$ is a vector potential corresponding to the IR laser pulses. Furthermore, we consider the following ansatz for the time-dependent wavefunction under an intense femtosecond-IR laser pulse and the weak attosecond-XUV laser pulse,

$$|\Psi(t)\rangle = |\Phi_{GS}\rangle + \sum_k c_k(t) \hat{a}^\dagger_{c,k+A_{IR}(t)/\hbar c} \hat{a}_{v,k+A_{IR}(t)/\hbar c} |\Phi_{gs}\rangle, \tag{30}$$

where $c_k(t)$ is an expansion coefficient. Here, the expansion coefficient for the ground state wavefunction $|\Phi_{GS}\rangle$ is fixed to 1, assuming that the excitation from the ground state is weak enough. By assuming that the weak attosecond-XUV laser pulse can only weakly excite the electronic ground state to electron-hole states by the dipole interaction, the equation of motion for the coefficient $c_k(t)$ is given by

$$i\hbar\dot{c}_k(t) = \left[\sum_{k'} H_{k,k'}(t)c_{k'}(t)\right] + E_{XUV}(t) \cdot D_{k+eA(t)/\hbar c}, \tag{31}$$

where $E_{XUV}(t)$ is the electric field of the attosecond-XUV laser pulse, and $D_k(t)$ is the transition dipole moment corresponding to the transition between the electronic ground state $|\Phi_{GS}\rangle$ and the electron-hole state $\hat{a}^\dagger_{c,k+A_{IR}(t)/\hbar c}\hat{a}_{v,k+A_{IR}(t)/\hbar c}|\Phi_{gs}\rangle$. In this work, we employ the following parametrization for the transition dipole moment [32, 33]

$$D_k = i\frac{p_{mom}}{E_g + \frac{\hbar^2 k^2}{2\mu}}, \tag{32}$$

where p_{mom} is the uniform transition momentum matrix. For simplicity, we set p_{mom} to 1 a.u. However, we note that the value of p_{mom} does not affect the results because this degree of freedom is absorbed by the parameter optimization in the next fitting procedure in Sec. 11.

11 Static reflectivity and parameter optimization

Here, we describe a numerical scheme to evaluate the static optical property of the material based on the Wannier-Mott model. Then, we introduce the fitting parameter optimization based on the comparison of the computed optical property and the experimental data.

To evaluate static optical properties, we first compute the electron dynamics under a weak XUV field, $E_{XUV}(t)$. Then, based on the time-evolving wave function $|\Psi(t)\rangle$ in Eq. (30), the induced current is evaluated as

$$J_{XUV}(t) = \frac{1}{\Omega}\langle\Psi(t)|\hat{J}|\Psi(t)\rangle = -2\frac{e \cdot p_{mom}}{m_e}\frac{\Delta k}{2\pi}\sum_k \mathrm{Re}\left[c_k(t)\right]. \tag{33}$$

Furthermore, based on the induced current dynamics, the optical conductivity can be evaluated as

$$\sigma_{exc}(\omega) = \frac{\tilde{J}(\omega)}{\tilde{E}_{XUV}(\omega)}, \tag{34}$$

where $\tilde{J}(\omega)$ and $\tilde{E}_{XUV}(\omega)$ are the Fourier transform of the current $J(t)$ and the XUV laser field $E_{XUV}(t)$, respectively. The Fourier transform is defined with the damping function as

$$\tilde{J}(\omega) = \int_{-\infty}^{\infty} dt e^{i\omega t} J(t) e^{-\frac{t}{\tau_d}}, \tag{35}$$

$$\tilde{E}_{XUV}(\omega) = \int_{-\infty}^{\infty} dt e^{i\omega t} E_{XUV}(t) e^{-\frac{t}{\tau_d}}, \tag{36}$$

where τ_d is the decay constant and it is set to 2.6 fs according to the experimental observation (see the main text). Moreover, based on the computed optical conductivity, the linear susceptibility can be evaluated as

$$\chi_{exc}(\omega) = i\frac{\sigma_{exc}(\omega)}{\omega}, \tag{37}$$

In order to analyze the experimental reflectivity, we model the dielectric function of MgF_2 with the excitonic susceptibility $\chi_{exc}(\omega)$ in Eq. (37), assuming the following form

$$\epsilon_{\mathrm{MgF_2}}(\omega) = \epsilon_{valence}(\omega) + 4\pi c\left[\chi_{ex}(\omega) + \frac{1}{3}\chi_{ex}(\omega + \Delta_{SO})\right], \tag{38}$$

where $\epsilon_{valence}$ is a complex dielectric function modeling the valence electron response, the parameter c determines the strength of the excitonic contribution. The effect of the spin-orbit splitting is taken into account by the linear combination of the shifted excitonic susceptibility $\chi_{exc}(\omega)$ with the spin-orbit split Δ_{SO} according to previous works [15]. We set the spin-orbit split Δ_{SO} to 0.44 eV and the ratio of the two contributions to $1/3$, according to what reported in Ref. [17]. Furthermore, we assume the following form for the valence contribution

$$\epsilon_{valence}(\omega) = \alpha + i\frac{\beta}{\omega - \omega_0}, \tag{39}$$

where α, β, and ω_0 are fitting parameters. We determine the fitting parameters, c, α, β, ω_0 so as to minimize the deviation from the experimental static reflectivity in the range between $51\,\mathrm{eV}$ and $55\,\mathrm{eV}$. More precisely, we compute the theoretical reflectivity $R_{theo}(\omega)$ from the dielectric function of Eq. (38) and compute the deviation from the experimental result

$$\Delta_{error} = \int_{\omega_i=51\text{ eV}}^{\omega_f=55\text{ eV}} d\omega \left| R_{exp}(\omega) - R_{theo}(\omega) \right|. \tag{40}$$

Then, we optimize the fitting parameters, c, α, β, ω_0 so as to minimize the error Δ_{error}. As shown in Fig. 1b in the main text, the model dielectric function with the optimized parameters shows very good agreement with the experimental result. Here, we obtained the following optimized parameters: $\alpha = 0.85$, $\beta = 2.3$ eV/$\hbar$, $c = 0.014$, and $\omega_0 = 45.6$ eV/$\hbar$. Note that, since the characteristic frequency ω_0 of $\epsilon_{valence}(\omega)$ is substantially smaller than the photon-energy range of interest (50-60 eV), the structures in the static reflectivity in Fig. 1b purely originate from the excitonic contribution $\chi_{exc}(\omega)$ based on the Wannier-Mott model.

12 Transient reflectivity with numerical pump-probe simulation

We next describe a theoretical scheme to investigate the transient optical property of MgF_2 with the model introduced in the previous section, 11. For this purpose, we employ the numerical pump-probe simulation [34]. Here, we compute the electron dynamics under both the IR vector potential $A_{IR}(t)$ and the XUV electric field $E_{XUV}(t)$, and the induced current is evaluated by Eq. (33). Based on the induced current $J_{XUV}(t)$, we evaluate the transient dielectric function with Eq. (38) through the Fourier transform of Eq. (35) and Eq. (36). Furthermore, the transient reflectivity is evaluated with the transient dielectric function. We repeat the numerical pump-probe simulation while changing the time-delay between the IR and XUV laser pulses. For the IR laser field, we employ the following form

$$A_{IR}(t) = -c\frac{E_{IR,0}}{\omega_{IR}} \left[\cos\left(\frac{\pi t}{T_{IR}} \right) \right]^2 \sin\left(\omega_{IR} t \right), \tag{41}$$

in the domain $-T_{IR}/2 < t < T_{IR}/2$ and zero outside. Here, $E_{IR,0}$ is a peak field strength of the IR pulse, ω_{IR} is a mean frequency of the pulse, and T_{IR} is its full duration. We set ω_{IR} to 1.55 eV/$\hbar$, and T_{IR} to 16 fs. The corresponding full width at half maximum is 6 fs, close to the value used in the experiment. As a corresponding attosecond XUV pulse, we employ the following form

$$E_{XUV}(t) = E_{XUV,0}\left[\cos\left(\frac{\pi(t+\tau_{delay})}{T_{XUV}}\right)\right]^4 \cos\left(\omega_{XUV}(t+\tau_{delay})\right), \quad (42)$$

in the domain $-T_{XUV}/2 < t + \tau_{delay} < T_{XUV}/2$ and zero outside. Here, τ_{delay} is the delay between the IR laser pulse and the XUV laser pulse. We set the mean frequency of the XUV pulse ω_{XUV} to 55 eV/$\hbar$, and the full duration T_{XUV} to 1 fs. The corresponding full width at half maximum is 260 as.

We perform the above numerical pump-probe simulation by setting the IR field strength $E_{IR,0}$ to 12.3 MV/cm inside the sample. The corresponding incident laser intensity in the vacuum is 10^{12} W/cm^2 under the incident angle of $73.5°$ and the MgF_2 refractive index of 1.375 at the photon energy of 1.55 eV [35]. Figure 10 presents the differential reflectivity as a function of photon energy and pump-probe delay as obtained with the full model described above. The $\Delta R/R$ reported in Fig. 10(a) shows a slow (Fig. 10(b)) and a fast (Fig. 10(c)) component,

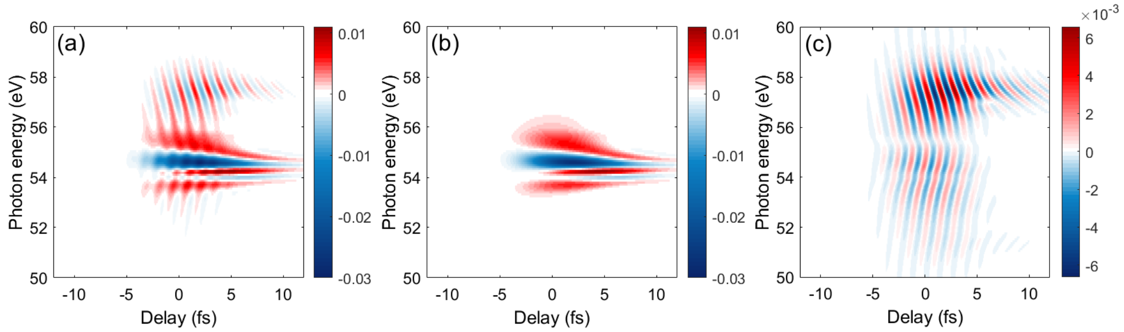

Figure 10: **(a)** Calculated $\Delta R/R$ spectrogram for the full model described in Sec. 11 and Sec. 12. (**b**), (**c**), associated slow and fast components, respectively.

described in detail in the main manuscript. In addition, it is possible to separate the upper and

lower transitions of the $L_{2,3}$ Mg edge (Fig. 11) by freezing the other contribution of χ_{exc} in Eq. (38), showing that the stronger contribution comes from the $2p_{1/2}$ state, which alone can describe all the main features of the full calculations.

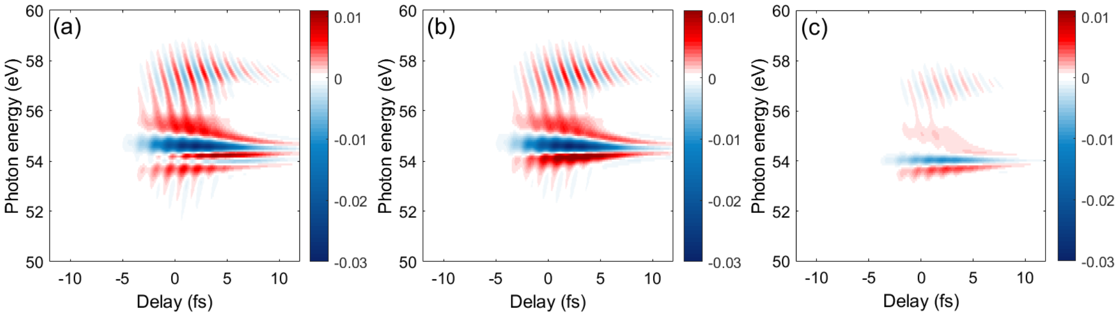

Figure 11: **(a)** Calculated $\Delta R/R$ spectrogram for the full model described in Sec. 11 and Sec. 12. (**b**), (**c**), associated contribution of the Mg $2p_{1/2}$ and Mg $2p_{3/2}$ states, respectively.

13 Pure exciton calculations

In order to investigate the atomic nature of the exciton in the transient reflection signal, we construct a *pure exciton* model based on the above Wannier-Mott model. The pure exciton model consists of the three levels: one is the electronic ground state $|\Psi_{GS}\rangle$, the second state is the excitonic ground state (bright exciton state), and the third state is the excitonic first excited state (dark exciton state) in Fig. 1c in the main text. The bright and dark excitonic states are computed by diagonalizing the Hamiltonian of the Wannier-Mott model, Eq. (26). Hence the pure exciton model is described by the following 3-by-3 Hamiltonian matrix

$$H_{ex}(t) = \begin{pmatrix} 0 & -e \cdot d_c \cdot E_{XUV}(t) & 0 \\ -e \cdot d_c \cdot E_{XUV}(t) & \epsilon_b & -e \cdot d_{exc} \cdot E_{IR}(t) \\ 0 & -e \cdot d_{exc} \cdot E_{IR}(t) & \epsilon_d \end{pmatrix}, \quad (43)$$

where ϵ_b and ϵ_d are the energy levels of the bright and dark excitonic states, respectively. Note that the energy level of the electronic ground state $|\Phi_{GS}\rangle$ is set to zero. The transition dipole moment matrix between the electronic ground state and the bright exciton states is denoted as

d_c while that between the bright and dark excitonic state is denoted as d_{exc}. Here, the XUV laser pulse $E_{XUV}(t)$ excites the electronic system from the electronic ground state to the bright exciton state while the IR laser pulse $E_{IR}(t)$ causes the transition between the bright and dark excitonic states. Since the model is constructed by the above Wannier-Mott model, the parameters of the Hamiltonian, Eq. (43), can be extracted from the model. The extracted parameters are as follows: $\epsilon_b = 54.4$ eV, $\epsilon_c = 55.7$ eV, and $d_{exc} = 3.5$ a.u. For simplicity, we set d_c to 1 a.u., but the choice of d_c does not affect the result because this degree of freedom is absorbed by the parameter optimization explained in Sec. 11.

The time evolution of the pure exciton model is described by the following quantum master equation

$$\frac{d}{dt}\rho(t) = \frac{[H_{ex}, \rho(t)]}{i\hbar} - \frac{1}{\tau_d}\begin{pmatrix} 0 & \rho_{12}(t) & 0 \\ \rho_{21}(t) & 0 & 0 \\ 0 & 0 & 0 \end{pmatrix}, \quad (44)$$

where $\rho(t)$ is the density matrix of the system, and $\rho_{ij}(t)$ is the i-j component of the density matrix. Here, we employ the simple relaxation time approximation for the coherence of the electronic ground state and the bright exciton state in order to naturally introduce the line broadening of the excitonic peak in the optical property. To be consistent with the above Wannier-Mott model analysis as well as the experimental finding, we set the relaxation time τ_d to 2.6 fs.

The optical property of the pure exciton model can be evaluated by a similar procedure as the full Wannier-Mott model described in the section 11. First we evaluate the induced polarization dynamics under the XUV laser field as

$$P_{ex}(t) = \mathrm{Tr}\left[\rho(t)\begin{pmatrix} 0 & -e\cdot d_c & 0 \\ -e\cdot d_c & 0 & 0 \\ 0 & 0 & 0 \end{pmatrix}\right]. \quad (45)$$

Then, we compute the susceptibility $\chi_{exc}(\omega)$ with the Fourier transform of the polarization and the XUV laser field as

$$\chi_{exc}(\omega) = \frac{\tilde{P}_{exc}(\omega)}{\tilde{E}_{exc}(\omega)}. \quad (46)$$

Note that we do not employ the damping function in the Fourier transform since the decoherence effect has been taken into account in the quantum master equation, Eq. (44). Finally, we model the dielectric function with Eq. (38). Here, we repeated the parameter optimization for the pure exciton model with the same procedure explained in Sec. 11, and we obtain the following optimized parameters: $\alpha = 0.84$, $\beta = 2.3$ eV/$\hbar$, $c = 1.9 \times 10^{-2}$, and $\omega_0 = 43.6$ eV//$\hbar$.

Based on the above pure exciton model, one can further evaluate the transient optical property under the presence of IR fields with the numerical pump-probe simulation (see Sec. 12). The results are reported in Fig. 12(a), together with the temporal decomposition in femtosecond and attosecond components (Figs. 12(b) and 12(c), respectively). It is possible to notice that,

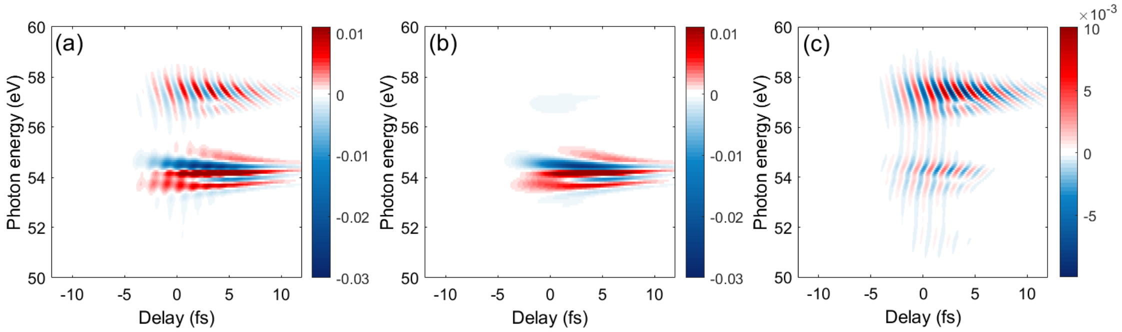

Figure 12: **(a)** Calculated $\Delta R/R$ spectrogram considering only the excitonic contribution. (**b**), (**c**), associated slow and fast components, respectively.

while the slow component of Fig. 12(b) is in good qualitative agreement with the slow component of the full results (Fig. 10(b)), the fast component differs significantly and fails to reproduce the V-shaped structure observed in the experiments (compare Fig. 12(c) and Fig. 10(c)).

14 Pure crystal calculations

Here, we investigate a bulk nature in the transient reflection signal. For this purpose, we evaluate the pure bulk contribution by eliminating the excitonic contribution from the full Wannier-Mott model described in Sec. 12. To eliminate the excitonic contribution, we set the electron-hole

interaction V_0 in Eq. (27) to zero. Then, we perform the same numerical pump-probe simulation with the parameterization in the section 12. The results are reported in Fig. 13(a), together with the temporal decomposition in femtosecond and attosecond components (Figs. 13(b) and (c), respectively). The pure crystal calculations show opposite behavior than the pure exciton results

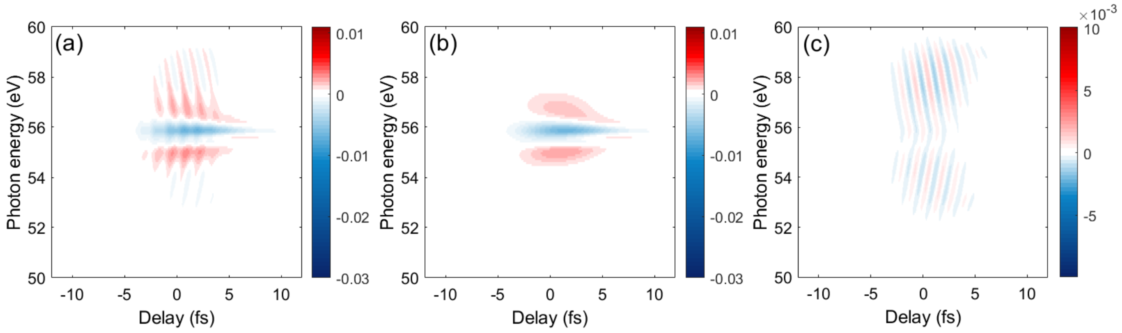

Figure 13: **(a)** Calculated $\Delta R/R$ spectrogram considering only the crystal without exciton formation. (**b**), (**c**), associated slow and fast components, respectively. In all the panels the color scales are kept the same as for Figs. 10 and 12.

described in the previous section. In this case indeed, the fast component (Fig. 13(b)) shows the same V-shaped structure, but shifted upwards in energy. The slow component (Fig. 13(c)), instead, cannot reproduce the dispersive profiles observed in the experimental results (see Fig. 2 in the main manuscript). Note that once the electron-hole interaction V_0 is set to zero in the Wannier-Mott model, the model is reduced to the parabolic two-band model used in Refs. [29, 15]. Hence, the transient response is dominated by the DFKE.

15 Real time dynamics of exciton dipole moment

To obtain further insight into the microscopic exciton dynamics, we evaluate the IR-induced dipole moment dynamics of the exciton. For this purpose, we compute the electron dynamics under the IR laser pulse by setting the initial wavefunction $|\Psi(t = -\infty)\rangle$ to the excitonic ground state (bright exciton state). To evaluate the dipole moment, we first define the IR-

induced current with the Wannier-Mott model. Here, the current operator $\hat{J}_{IR}$ is given by

$$\hat{J}_{IR} = -c\frac{\partial H_{k,k'}(t)}{\partial A_{IR}}. \tag{47}$$

Then, one can evaluate the IR-induced current as

$$J(t) = \langle \Psi(t)|\hat{J}|\Psi(t)\rangle. \tag{48}$$

Furthermore, the corresponding polarization can be evaluated by the time-integration as

$$P(t) = D_{exc}(t) = \int_{-\infty}^{t} dt' J(t'). \tag{49}$$

Note that, in the Wannier-Mott model, the induced polarization can be seen as the induced dipole moment of the exciton in the real-space expression. Thus, we treat it as the exciton dipole moment hereafter.

In order to evaluate the phase delay between the IR electric field and the exciton dipole oscillations $D_{exc}(t)$ we proceeded as indicated in Sec. 7. We concentrate on the time window of temporal overlap (between -8.5 and +8.5 fs) and perform the Fourier transform of the dipole only in this region. Furthermore, we squared both E_{IR} and the excitonic dipole in order to evaluate the phase difference at the same frequency $2\omega_{IR}$ used in the case of $\Delta R/R$. Figure 14 shows the results which are summarized in Figs. 4D and 4E of the main manuscript.

16 Spin-orbit splitting

As discussed in the previous sections, the spin-orbit splitting of the Mg $2p$ state is responsible for the double peak observed in the static reflectivity. As a results, the transient reflectivity trace is composed by two partially overlapping signals, one involving the promotion of a $2p_{1/2}$ electron and the other a $2p_{3/2}$ electron. Figure 15 shows the phase delays extracted from the full model calculations (crystal plus exciton) and considering only the $2p_{1/2}$ contribution (blue curve), the $2p_{3/2}$ contribution (green curve), or both (red curve). Since the signal from the $2p_{1/2}$

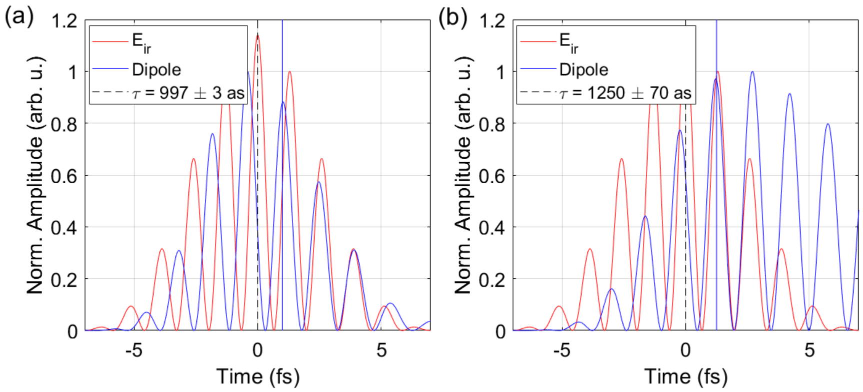

Figure 14: **(a)** blue curve, excitonic dipole for the full calculations. Red curve, IR electric field squared. The distance between two maxima, underlined by the vertical black dashed and blue solid lines, visualizes the phase delay. **(b)** Same as in (a) but for the exciton-only calculations.

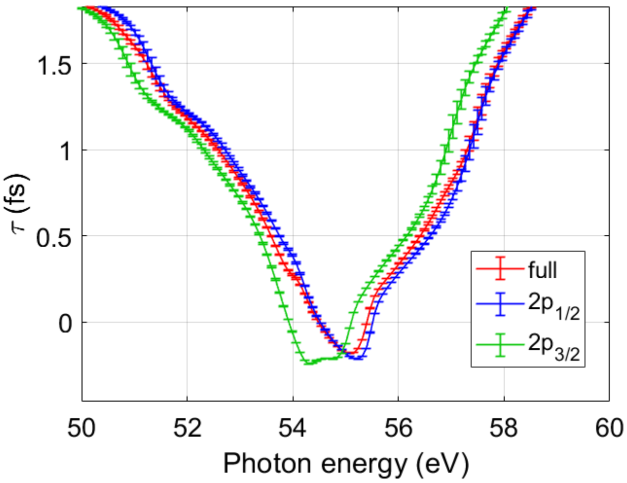

Figure 15: Phase delays calculated with the full model (crystal plus exciton, same as Fig. 3c of the main manuscript) and separated in the upper (green curve) and lower (blue curve) state contribution. As it is possible to observe the full calculations resemble the strongest transition ($2p_{1/2}$)

state is stronger than the one involving the $2p_{3/2}$ state, the full response is largely dominated by the former. We notice that the phase delay which involves a different $2p$ hole is not identical, but the difference is too little to be observed with our current experimental setup. Therefore, the exact description of the spin-orbit coupling is not fundamental to our analysis, as both the green and blue curve of Fig. 15 fall within the experimental uncertainty. The main effect of considering the spin orbit splitting is to achieve a better agreement between the experimental 2D maps and the calculated ones, without changing the Physics under examination.

17 IR intensity dependence

In order to verify the robustness of our results against the IR pulse intensity we performed the calculations for four values of maximum electric field inside the sample, 2.9, 9.26, 12.3 and 34.7 MV/cm, corresponding to an IR intensity outside the sample of 5.7×10^{10}, 5.7×10^{11}, 1×10^{12} and 8×10^{12} W/cm^2. The differential reflectivity spectrograms calculated for increasing IR peak intensity are reported in Fig. 16. We found $\Delta R/R$ to be mostly in the linear regime, showing appreciable modifications only for the strongest intensity $I_{IR} = 8 \times 10^{12}$ W/cm^2. The same behavior is observed in the phase delay τ. Figure 17 shows the phase delay extracted for the calculations reported in Fig. 16 considering the full model (Fig. 17(a)), only the exciton (Fig. 17(b)) or the crystal (Fig. 17(c)) contribution. The curves are nearly identical, with small deviations only for $I_{IR} = 8 \times 10^{12}$ W/cm^2 (orange curve). This demonstrates that the observed features and the attosecond and femtosecond dynamics discussed in the main manuscript are robust with the respect to a wide range of IR intensities.

18 Binding energy dependence

In order to investigate the role of exciton localization, we used the full model of Sec. 12 to calculate $\Delta R/R$ while changing the exciton binding energy E_b between 0.7 and 4.2 eV by ma-

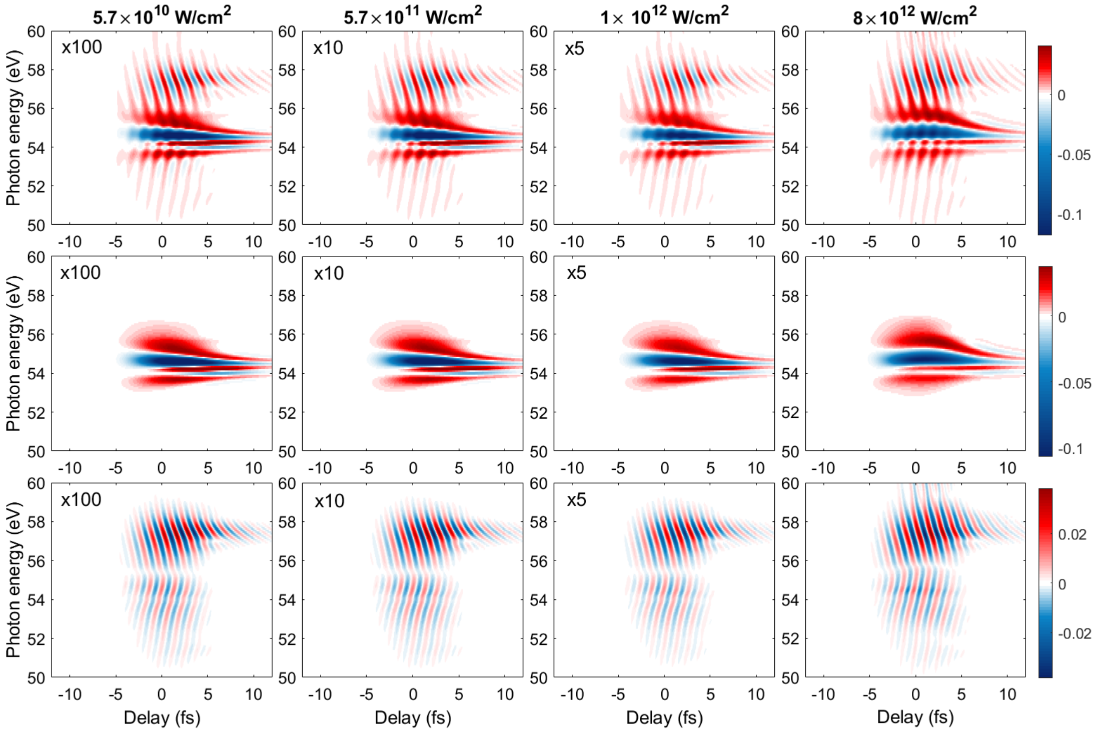

Figure 16: Differential reflectivity spectrograms calculated for different IR intensities. The first raw presents the full calculations. Second and third rows are the associated slow and fast components. Each column corresponds to a different IR peak intensity, reported in the relative title.

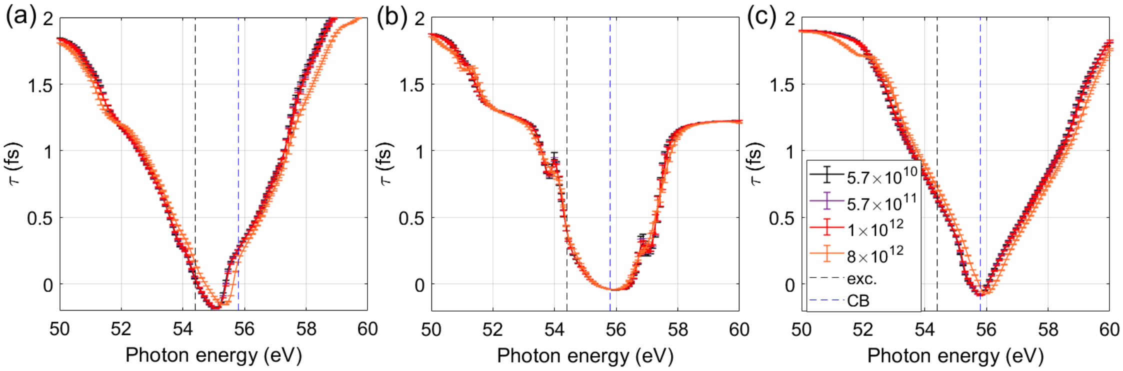

Figure 17: Phase delay τ, calculated for the full system **(a)**, the pure quasi-particle **(b)** or the pure crystal **(c)**. In all panels, black, violet, red and orange curves correspond to an IR peak intensity I_{IR} equal to 5.7×10^{10}, 5.7×10^{11}, 1×10^{12} and 8×10^{12} W/cm^2. The legend is reported in (c).

nipulating the electron-hole interaction strength V_0. The results calculated for an IR intensity of 1×10^{12} W/cm^2 are displayed in Fig. 18. As it is possible to notice, $\Delta R/R$ is strongly af-

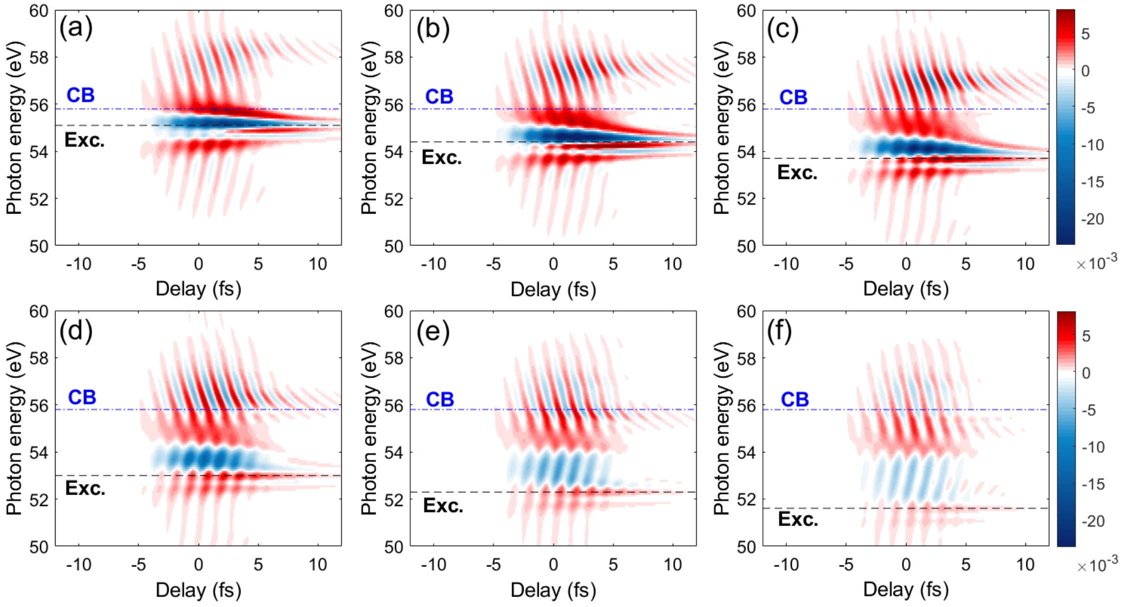

Figure 18: **(a)** Transient reflectivity spectrogram calculated with the full model described in Sec. 12 using an exciton binding energy $E_b = 0.7$ eV. The horizontal blue dash-dotted line marks the conduction band bottom, the black dashed line indicates instead the excitonic transition. **(b)-(f)**, same as in (a), but with E_b equal to 1.4, 2.1, 2.8, 3.5, 4.2 eV, respectively.

fected by the value of E_b, which modifies the amplitude and shape of the femtosecond transient features. The effect on the attosecond dynamics is described and summarized in Fig 4 of the main manuscript: the phase delay τ exhibits an almost rigid energy shift, moving towards lower photon energies for an increasing E_b. In order to study the relation between E_b and the energy shift experienced by τ, we calculated the quantity ΔE which minimized the distance between the energy dependent phase delay of the pure crystal $\tau_{cry}(E)$ and the energy dependent phase delay of the full system with a given value of E_b, $\tau_{E_b}(E)$, defined as follows:

$$\Delta\tau(E, \Delta E) = \sqrt{\frac{\int_{E_1}^{E_2} |\tau_{cry}(E) - \tau_{E_b}(E - \Delta E)|^2 \, dE}{E_2 - E_1}} \tag{50}$$

The results for $E_1 = 46$ eV and $E_2 = 63$ eV are reported in Fig. 19. One can notice that even if

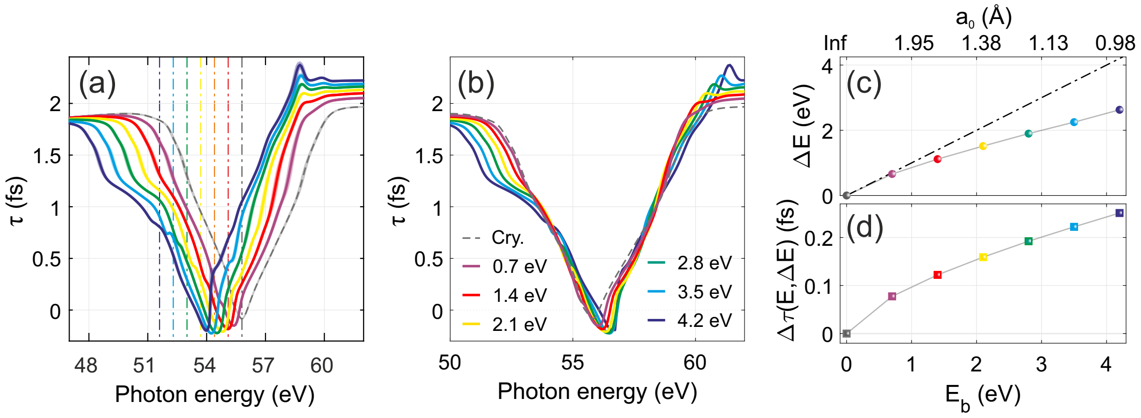

Figure 19: **(a)** Behavior of the calculated phase delay τ as a function of the exciton binding energy E_b (legend in (b)). The vertical dash-dotted lines mark the exciton position. Same data as in Fig. 4c of the main manuscript. **(b)** Phase delays from (a) (same color coding) shifted in energy by a quantity ΔE, in order to minimize the distance from the pure crystal response (dashed gray curve). **(c)** ΔE as a function of E_b (Bohr radius a_0), showing a deviation from the linear behavior (black dot-dashed line). Same data as in Fig. 4e of the main manuscript. **(d)** Distance between the energy dependent phase delay of the pure crystal $\tau_{cry}(E)$ and the energy dependent phase delay of the full system with a given value of E_b, defined in eq. (50) and calculated for the energy shifts ΔE reported in (c).

the overall V shape of the phase delay τ is preserved for all the values of E_b considered, we are not observing a rigid shift of the bare crystal curve. The energy shift ΔE which minimizes the residual distance between the translated curves does not scale linearly with the binding energy (Fig. 19(c)) and the distance between the curves is not constant (Fig. 19(d)).

It is worth noting that the exciton binding energy is related to its degree of localization. Indeed, for an hydronic quasi-particle, there is a precise relation between its Bohr radius, a_0 and its binding energy E_b, given by the following formula [36, 37]:

$$E_b = -\frac{\hbar^2}{2\mu a_0^2}, \tag{51}$$

where μ is the reduced mass of the electron-hole system. For the case of MgF_2, expressing the binding energy in eV and the Bohr radius in Ångströms, we obtain:

$$E_b = \left[\frac{1.952}{a_0}\right]^2. \tag{52}$$

19 MgF_2 valence exciton

The physical mechanisms based on intra-band motion which defines the sub-fs optical response of the system is not an exclusive characteristic of core excitons, but is expected to happen also in technologically more relevant valence excitons, as long as they are Wannier-Mott excitons [36]. Whether an experimental investigation of the attosecond response of valence excitons is demanding, they can be theoretically studied using the model reported here. In this respect, MgF_2 is a perfect candidate as it exhibits a strong excitonic feature close to its conduction band, associated with a valence exciton [38]. Compared to the core exciton, the valence exciton is characterized by a weaker binding energy (about 500 meV) and longer life times (in the ps/ns regime [39]). Therefore an analysis of the slow component of the transient optical properties will show an almost constant signal in the pump-probe time window under examination (about 30 fs). The sub-fs response is instead in agreement with what found for core excitons. Figure 20 shows a comparison between the phase delay as extracted from the imaginary part of the differential conductivity $\Delta\sigma$ calculated for the core exciton (Fig. 20(a)) and a valence exciton characterized by a binding energy of 500 meV (Fig. 20(b)) or 1 eV (Fig. 20(c)). To model the valence exciton we assumed the CB bottom to be located 12.17 eV above the VB [38].

At first we observe that the phase delay of $Im\{\Delta\sigma\}$ is strictly related to the phase delay observed in the differential reflectivity trace (compare Fig. 20(a) with the Fig. 4c in the main manuscript). Furthermore, also for the valence exciton, the full system response has a V-shaped dispersion which is closely related to the pure crystal contribution (DFKE) and which is not correctly reproduced by an atomic-like model where the field interaction is dominated by the optical Stark shift (pure exciton case). Furthermore, also in case of valence excitons the phase delay evaluated at the exciton transition, τ_{ex}, or at the CB position, τ_{CB}, change with the exciton binding energy E_b. Figure 21 shows the equivalent of Fig. 4f of the main manuscript, but extracted from the valence exciton calculations reported in Figs. 20(b) and 20(c).

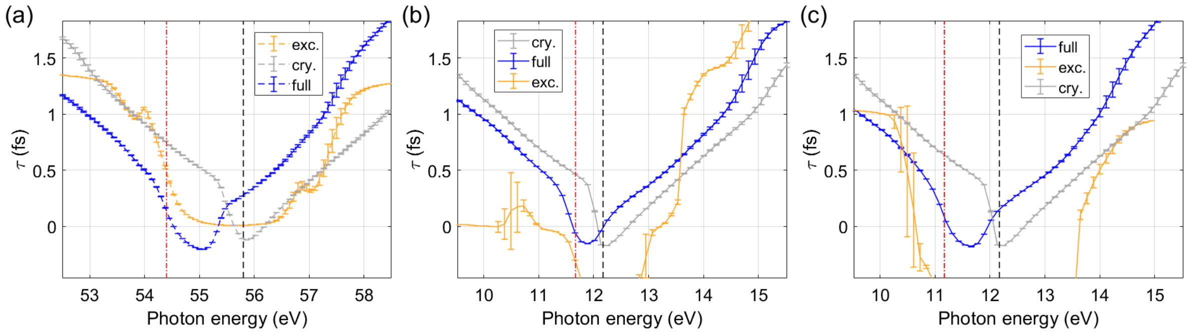

Figure 20: **(a)** Phase delays extracted from the sample differential conductivity, $\Delta\sigma$, calculated with the full model (blue), the pure exciton (orange) and the pure crystal (gray). **(b)**, **(c)**, Same quantities calculated for a valence exciton with 500 meV or 1 eV binding energy, respectively. In the three panels, the black dashed vertical line indicates the CB position while the red dot-dashed line indicates the excitonic resonance.

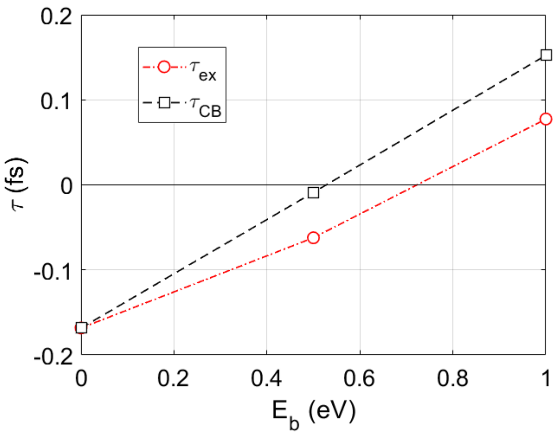

Figure 21: **(a)** Value of the phase delay τ evaluated at the valence exciton vertical transition (τ_{ex}, red open circles), or at the bottom of the CB (τ_{CB}, black open squares) for the two values of E_b considered.

We note that the absolute values of τ_{CB} and τ_{ex} reported in Fig. 21 cannot be directly compared with the values reported in Fig. 4f of the main manuscript since they have been extracted from the differential conductivity and not from the differential reflectivity. Nevertheless, we observe the same qualitative behavior: τ_{CB} and τ_{ex} can be controlled acting on the exciton binding energy.

Summarizing the above considerations, we can conclude that our results are not limited to core excitons, but can hold also for valence excitons, under the assumption that the quasi-particle can be described by the Wannier-Mott model. The situation could be different for Frenkel excitons, which have a different physical origin and for which we expect the intra-band contribution to be less dominant. As a consequence, the full system response might not follow the V-shaped dispersion.

References

[1] G. D. Lucarelli, B. Moio, G. Inzani, N. Fabris, L. Moscardi, F. Frassetto, L. Poletto, M. Nisoli, and M. Lucchini, "Novel beamline for attosecond transient reflection spectroscopy in a sequential two-foci geometry," Rev. Sci. Instrum., vol. 91, no. 5, p. 053002, 2020.

[2] M. Nisoli, S. De Silvestri, and O. Svelto, "Generation of high energy 10 fs pulses by a new pulse compression technique," Applied Physics Letters, vol. 68, no. 20, pp. 2793–2795, 1996.

[3] C. Feng, J.-F. Hergott, P.-M. Paul, X. Chen, O. Tcherbakoff, M. Comte, O. Gobert, M. Reduzzi, F. Calegari, C. Manzoni, M. Nisoli, and G. Sansone, "Complete analog control of the carrier-envelope-phase of a high-power laser amplifier," Opt. Express, vol. 21, pp. 25248–25256, Oct 2013.

[4] F. Ferrari, F. Calegari, M. Lucchini, C. Vozzi, S. Stagira, G. Sansone, and M. Nisoli, "High-energy isolated attosecond pulses generated by above-saturation few-cycle fields," Nature Photonics, vol. 4, pp. 875–879, dec 2010.

[5] Jouanin, C., Albert, J.P., and Gout, C., "Band structure and optical properties of magnesium fluoride," J. Phys. France, vol. 37, no. 5, pp. 595–602, 1976.

[6] W. F. Hanson, E. T. Arakawa, and M. W. Williams, "Optical properties of mgo and mgf2 in the extreme ultraviolet region," Journal of Applied Physics, vol. 43, no. 4, pp. 1661–1665, 1972.

[7] J. Itatani, F. Quéré, G. L. Yudin, M. Y. Ivanov, F. Krausz, and P. B. Corkum, "Attosecond streak camera," Phys. Rev. Lett., vol. 88, p. 173903, Apr 2002.

[8] Y. Mairesse and F. Quéré, "Frequency-resolved optical gating for complete reconstruction of attosecond bursts," Physical Review A, vol. 71, p. 011401, jan 2005.

[9] C. J. Kaplan, P. M. Kraus, A. D. Ross, M. Zürch, S. K. Cushing, M. F. Jager, H.-T. Chang, E. M. Gullikson, D. M. Neumark, and S. R. Leone, "Femtosecond tracking of carrier relaxation in germanium with extreme ultraviolet transient reflectivity," Phys. Rev. B, vol. 97, p. 205202, May 2018.

[10] C. J. Kaplan, P. M. Kraus, E. M. Gullikson, L. J. Borja, S. K. Cushing, M. Zürch, H.-T. Chang, D. M. Neumark, and S. R. Leone, "Retrieval of the complex-valued refractive index of germanium near the M 4,5 absorption edge," Journal of the Optical Society of America B, vol. 36, p. 1716, jun 2019.

[11] D. Attwood, Soft X-Rays and Extreme Ultraviolet Radiation: Principles and Applications. Cambridge University Press, 1999.

[12] W. S. M. Werner, K. Glantschnig, and C. Ambrosch-Draxl, "Optical constants and inelastic electron-scattering data for 17 elemental metals," Journal of Physical and Chemical Reference Data, vol. 38, no. 4, pp. 1013–1092, 2009.

[13] M. Lucchini, S. A. Sato, F. Schlaepfer, K. Yabana, L. Gallmann, A. Rubio, and U. Keller, "Attosecond timing of the dynamical Franz-Keldysh effect," Journal of Physics: Photonics, jan 2020.

[14] J. Yakovlev and V. S. Gagnon, "The direct evaluation of attosecond chirp from a streaking measurement," Appl Phys B, pp. 303–309, 2011.

[15] F. Schlaepfer, M. Lucchini, S. A. Sato, M. Volkov, L. Kasmi, N. Hartmann, A. Rubio, L. Gallmann, and U. Keller, "Attosecond optical-field-enhanced carrier injection into the GaAs conduction band," Nature Physics, vol. 14, pp. 560–564, jun 2018.

[16] F. Schlaepfer, A. Ludwig, M. Lucchini, L. Kasmi, M. Volkov, L. Gallmann, and U. Keller, "Gouy phase shift for annular beam profiles in attosecond experiments," Optics Express, vol. 25, p. 3646, feb 2017.

[17] P. Rabe, B. Sonntag, T. Sagawa, and R. Haensel, "The optical absorption of mgf2, mgcl2, and mgbr2 in the vicinity of the mg l-shell transitions," physica status solidi (b), vol. 50, no. 2, pp. 559–569, 1972.

[18] R. Géneaux, C. J. Kaplan, L. Yue, A. D. Ross, J. E. Bækhøj, P. M. Kraus, H.-T. Chang, A. Guggenmos, M.-Y. Huang, M. Zürch, K. J. Schafer, D. M. Neumark, M. B. Gaarde, and S. R. Leone, "Attosecond time-domain measurement of core-level-exciton decay in magnesium oxide," Phys. Rev. Lett., vol. 124, p. 207401, May 2020.

[19] A. Moulet, J. B. Bertrand, T. Klostermann, A. Guggenmos, N. Karpowicz, and E. Goulielmakis, "Soft x-ray excitonics," Science, vol. 357, no. 6356, pp. 1134–1138, 2017.

[20] G. D. Mahan, "Photoemission from alkali halides: Energies and line shapes," Phys. Rev. B, vol. 21, pp. 4791–4803, May 1980.

[21] G. D. Mahan, "Emission spectra and phonon relaxation," Physical Review B, vol. 15, no. 10, p. 4587, 1977.

[22] O. Aita, K. Ichikawa, and K. Tsutsumi, "Decay process of the mg 2p core exciton in magnesium halides studied by photoelectron spectroscopy," Phys. Rev. B, vol. 39, pp. 10266–10274, May 1989.

[23] G. H. Wannier, "The structure of electronic excitation levels in insulating crystals," Phys. Rev., vol. 52, pp. 191–197, Aug 1937.

[24] N. F. Mott, "Conduction in polar crystals. ii. the conduction band and ultra-violet absorption of alkali-halide crystals," Trans. Faraday Soc., vol. 34, pp. 500–506, 1938.

[25] F. Fuchs, C. Rödl, A. Schleife, and F. Bechstedt, "Efficient $\mathrm{O}(N^2)$ approach to solve the bethe-salpeter equation for excitonic bound states," Phys. Rev. B, vol. 78, p. 085103, Aug 2008.

[26] A. Schiffrin, T. Paasch-Colberg, N. Karpowicz, V. Apalkov, D. Gerster, S. Mühlbrandt, M. Korbman, J. Reichert, M. Schultze, S. Holzner, J. V. Barth, R. Kienberger, R. Ernstorfer, V. S. Yakovlev, M. I. Stockman, and F. Krausz, "Optical-field-induced current in dielectrics," Nature, vol. 493, pp. 70–74, Jan 2013.

[27] T. T. Luu, M. Garg, S. Y. Kruchinin, A. Moulet, M. T. Hassan, and E. Goulielmakis, "Extreme ultraviolet high-harmonic spectroscopy of solids," Nature, vol. 521, pp. 498–502, May 2015.

[28] E. N. Osika, A. Chacón, L. Ortmann, N. Suárez, J. A. Pérez-Hernández, B. Szafran, M. F. Ciappina, F. Sols, A. S. Landsman, and M. Lewenstein, "Wannier-bloch approach to localization in high-harmonics generation in solids," Phys. Rev. X, vol. 7, p. 021017, May 2017.

[29] M. Lucchini, S. A. Sato, A. Ludwig, J. Herrmann, M. Volkov, L. Kasmi, Y. Shinohara, K. Yabana, L. Gallmann, and U. Keller, "Attosecond dynamical Franz-Keldysh effect in polycrystalline diamond," Science, vol. 353, pp. 916–919, aug 2016.

[30] S. A. Sato, H. Hübener, U. De Giovannini, and A. Rubio, "Ab initio simulation of attosecond transient absorption spectroscopy in two-dimensional materials," Applied Sciences, vol. 8, no. 10, p. 1777, 2018.

[31] A. D. Becke and E. R. Johnson, "A simple effective potential for exchange," The Journal of Chemical Physics, vol. 124, no. 22, p. 221101, 2006.

[32] J. B. Krieger and G. J. Iafrate, "Time evolution of bloch electrons in a homogeneous electric field," Phys. Rev. B, vol. 33, pp. 5494–5500, Apr 1986.

[33] S. A. Sato, M. Lucchini, M. Volkov, F. Schlaepfer, L. Gallmann, U. Keller, and A. Rubio, "Role of intraband transitions in photocarrier generation," Phys. Rev. B, vol. 98, p. 035202, Jul 2018.

[34] S. A. Sato, K. Yabana, Y. Shinohara, T. Otobe, and G. F. Bertsch, "Numerical pump-probe experiments of laser-excited silicon in nonequilibrium phase," Phys. Rev. B, vol. 89, p. 064304, Feb 2014.

[35] M. J. Dodge, "Refractive properties of magnesium fluoride," Appl. Opt., vol. 23, pp. 1980–1985, Jun 1980.

[36] G. L. Rocca, "Wannier–mott excitons in semiconductors," in Electronic Excitations in Organic Nanostructures, vol. 31 of Thin Films and Nanostructures, pp. 97 – 128, Academic Press, 2003.

[37] J. Jadczak, T. Smo, P. Kossacki, Y. S. Huang, and L. Bryja, "Exciton binding energy and hydrogenic Rydberg series in layered ReS 2," Sci. Rep., vol. 9, no. December 2018, p. 1578, 2019.

[38] Z. Yi and R. Jia, "Quasiparticle band structures and optical properties of magnesium fluoride," Journal of Physics: Condensed Matter, vol. 24, p. 085602, jan 2012.

[39] V. N. Kolobanov, V. V. Mikhailin, S. P. Chernov, D. A. Spassky, V. N. Makhov, M. Kirm, E. Feldbach, and S. Vielhauer, "Luminescence of singlet self-trapped excitons in MgF2," Journal of Physics: Condensed Matter, vol. 21, p. 375501, aug 2009.
